# Comparative genomics reveals *Cyclospora cayetanensis* possesses coccidia-like metabolism and invasion components but unique surface antigens

**DOI:** 10.1186/s12864-016-2632-3

**Published:** 2016-04-30

**Authors:** Shiyou Liu, Lin Wang, Huajun Zheng, Zhixiao Xu, Dawn M. Roellig, Na Li, Michael A. Frace, Kevin Tang, Michael J. Arrowood, Delynn M. Moss, Longxian Zhang, Yaoyu Feng, Lihua Xiao

**Affiliations:** State Key Laboratory of Bioreactor Engineering, School of Resources and Environmental Engineering, East China University of Science and Technology, Shanghai, 200237 China; Division of Foodborne, Waterborne, and Environmental Diseases, Centers for Disease Control and Prevention, Atlanta, GA 30333 USA; Shanghai–Ministry of Science and Technology Key Laboratory of Health and Disease Genomics, Chinese National Human Genome Center at Shanghai, 250 Bibo Road, Shanghai, 201203 China; Division of Scientific Resources, Centers for Disease Control and Prevention, Atlanta, GA 30333 USA; College of Animal Science and Veterinary Medicine, Henan Agricultural University, Zhengzhou, 450002 China

**Keywords:** *Cyclospora*, Genomics, Genome, Genetics, Evolution, Apicomplexan

## Abstract

**Background:**

*Cyclospora cayetanensis* is an apicomplexan that causes diarrhea in humans. The investigation of foodborne outbreaks of cyclosporiasis has been hampered by a lack of genetic data and poor understanding of pathogen biology. In this study we sequenced the genome of *C. cayetanensis* and inferred its metabolism and invasion components based on comparative genomic analysis.

**Results:**

The genome organization, metabolic capabilities and potential invasion mechanism of *C. cayetanensis* are very similar to those of *Eimeria tenella*. Propanoyl-CoA degradation, GPI anchor biosynthesis, and *N*-glycosylation are some apparent metabolic differences between *C. cayetanensis* and *E. tenella*. Unlike *Eimeria* spp., there are no active LTR-retrotransposons identified in *C. cayetanensis*. The similar repertoire of host cell invasion-related proteins possessed by all coccidia suggests that *C. cayetanensis* has an invasion process similar to the one in *T. gondii* and *E. tenella*. However, the significant reduction in the number of identifiable rhoptry protein kinases, phosphatases and serine protease inhibitors indicates that monoxenous coccidia, especially *C. cayetanensis,* have limited capabilities or use a different system to regulate host cell nuclear activities. *C. cayetanensis* does not possess any cluster of genes encoding the TA4-type SAG surface antigens seen in *E. tenella*, and may use a different family of surface antigens in initial host cell interactions.

**Conclusions:**

Our findings indicate that *C. cayetanensis* possesses coccidia-like metabolism and invasion components but unique surface antigens. Amino acid metabolism and post-translation modifications of proteins are some major differences between *C. cayetanensis* and other apicomplexans. The whole genome sequence data of *C. cayetanensis* improve our understanding of the biology and evolution of this major foodborne pathogen and facilitate the development of intervention measures and advanced diagnostic tools.

**Electronic supplementary material:**

The online version of this article (doi:10.1186/s12864-016-2632-3) contains supplementary material, which is available to authorized users.

## Background

*Cyclospora cayetanensis* is an emerging apicomplexan parasite related to *Eimeria* spp. [1]. After ingestion of food or water contaminated by oocysts, humans develop watery diarrhea, nausea and abdominal pain. In industrialized nations cyclosporiasis is often associated with travel to developing countries or outbreaks due to consumption of imported fresh produce [1, 2]. Since 2013, large multistate outbreaks of cyclosporiasis have occurred yearly in the United States and Canada, but outbreak investigations have been hampered by the lack of molecular diagnostic tools for trace-back studies [3] (http://www.cdc.gov/parasites/cyclosporiasis/outbreaks/2015/index.html).

The life cycle of *C. cayetanensis* is typical of monoxenous coccidia, which complete asexual and sexual development within a single host. Similar to *Eimeria* spp., *C. cayetanensis* probably has strict host specificity, infecting only enterocytes of humans. In contrast, another well-studied coccidian parasite, *Toxoplasma gondii*, has a heteroxenous life cycle, infecting not only enterocytes of its feline definitive hosts but also multiple tissues of various intermediate hosts, including humans [4]. The molecular determinants of host specificity and tissue tropism in apicomplexan parasites are poorly understood. Nevertheless, the host cell invasion mechanism of *T. gondii* has been studied extensively. Three essential secretory organelles, including micronemes and rhoptries of the apical complex and dense granules, are involved in the invasion process [5]. Before host cell invasion, apicomplexan sporozoites move across substrates by gliding, which is powered by an actin-myosin motor. The invasion begins with the secretion of several groups of proteins from micronemes, such as the apical membrane antigen 1 (AMA1) and rhoptry neck proteins (RONs), such as RON2, RON4 and RON5, forming a moving junction that is attached to the host cell cytoskeleton. This leads to the formation of numerous host-pathogen adhesion complexes consisting of microneme proteins (MICs) and surface antigens [6]. The parasite then moves across host membranes and develops a parasitophorous vacuole (PV) inside the host cell, where it grows and replicates. To evade the host immune system and survive in the intracellular environment, another large group of rhoptry proteins (ROPs) are delivered to the periplasmic surface of the PV and host cell nucleus, modulating host cell signaling pathways and gene expression [7, 8]. Some proteins secreted from dense granules (GRAs) are also involved in the regulation of host cell nuclear activities [9].

Few data exist on genetics of *C. cayetanensis*. To generate much needed sequence data and improve our understanding of its biology, we sequenced the genome of an isolate of *C. cayetanensis* and conducted a comparative genomic analysis. The results show that *C. cayetanensis* and *E. tenella* have similar genomic features and metabolic capabilities. They probably use a host cell invasion system similar to that in *T. gondii*, but a divergent system in modulating host cell signaling pathways. The specific surface antigens possessed by different coccidia may be the primary determinants for their host specificity.

## Results

### Genome sequencing and general features

We obtained 120.9 million of 100-bp paired-end reads from Illumina sequencing and 960,078 reads of 400-450 bp from Roche GS-FLX 454 sequencing, yielding over 200-fold coverage of the genome. A total of 4811 contigs with an overall length of 46,816,962 bp were generated in the *de novo* assembly of sequences (Additional file 1: Figure S1). After BLASTN analysis to eliminate contaminants from bacteria, Archaea, or host DNA, we obtained a draft genome of *C. cayetanensis* with a total length of 44,034,550 bp, a mean contig length of 19,170 bp, and an N50 contig of 61,202 bp (Additional file 2: Table S1). The genome of *C. cayetanensis* is slightly smaller than genomes of *T. gondii* and *E. tenella* (Table 1). The completeness of the draft genome of *C. cayetanensis* was estimated by using the BUSCO software (Additional file 3: Table S2). Altogether, 74.4 % of the core eukaryotic protein-encoding genes were covered by the genome of *C. cayetanensis,* which is comparable to that of whole genome sequences from *T. gondii* (85.1 %) and *E. tenella* (68.1 %). It has a gene density that is similar to that of *E. tenella* and *T. gondii*, but lower than that seen in some other apicomplexan parasites. In BLASTN analysis, we have identified the full mitochondrial and apicoplast genomes of *C. cayetanensis* [10].

**Table 1:** Comparison of genomic features of *Cyclospora cayetanensis* (Ccay) and other apicomplexan parasites^a^

Category	Cpar	Pfal	Bbov	Tgon	Eten	Ccay
No. of chromosomes	8	14	4	14	14	-
Total length of assembly (Mb)	9.10	22.85	8.18	65.67	51.86	44.03
No. of super contigs	8	16	14	2,263	4,664	2,297
GC content (%)	30.3	20.0	41.5	48.5	52.5	51.8
No. of genes	3,805	5,542	3,706	8,322	8,597	7,457
Total length of CDS (Mb)	6.83	12.58	5.58	20.03	13.05	11.92
GC content in CDS (%)	31.9	25.0	43.7	56.0	58.1	55.8
Mean length of genes (bp)	1,720	2,271	1,506	2,407	1,518	1,599
Gene density (genes/Mb)	418.1	242.5	453.1	126.7	165.8	169.4
Percent coding (%)	75.0	55.1	68.2	30.5	25.2	27.1
No. of genes with intron	163	3,055	2,241	6,729	6,563	6,358
% genes with introns	4.2	55.1	60.5	80.9	76.3	85.3
No. of tRNA	45	72	70	174	-	144
No. of tRNA^Met^	2	2	4	8	-	7
No. of rRNA^b^	15	28	-	420	4	11
No. of proteins with signal peptide	397	638	350	759	775	538
No. of proteins with apicoplast targeting signal	(22)	189	99	148	182	105
No. of proteins with transmembrane domain	832	1,754	677	1,103	1,378	1,247
No. of proteins with GPI-anchor	63	62	51	255	371	225
Apicoplast genome size (bp)	-	34,682	33,351	34,996	34,750	34,155
Mitochondrial genome size (bp)	-	5,967	6,005	~6,000^c^	6,213	6,229

^a^Sources of data: *Cryptosporidium parvum* (Cpar): CryptoDB release-6.0; *Plasmodium falciparum* (Pfal): PlasmoDB release-11.1; *Babesia bovis* (Bbov): PiroplasmaDB release-5.1; *Toxoplasma gondii* (Tgon): ToxoDB release-11.0; *Eimeria tenella* (Eten): ToxoDB release-11.0. Data on proteins with signal peptides, apicoplast targeting signal peptides and GPI-anchors were based on calculations using software specified in Methods. Dashes indicate the lack of data (for *E. tenella*) or the absence of organelles (for *C. parvum*)

^b^Based on annotation; actual numbers are greater due to the repetitive nature of the rRNA unit

^c^Based on Seeber et al. (2014) [72]

**Fig. 1:**
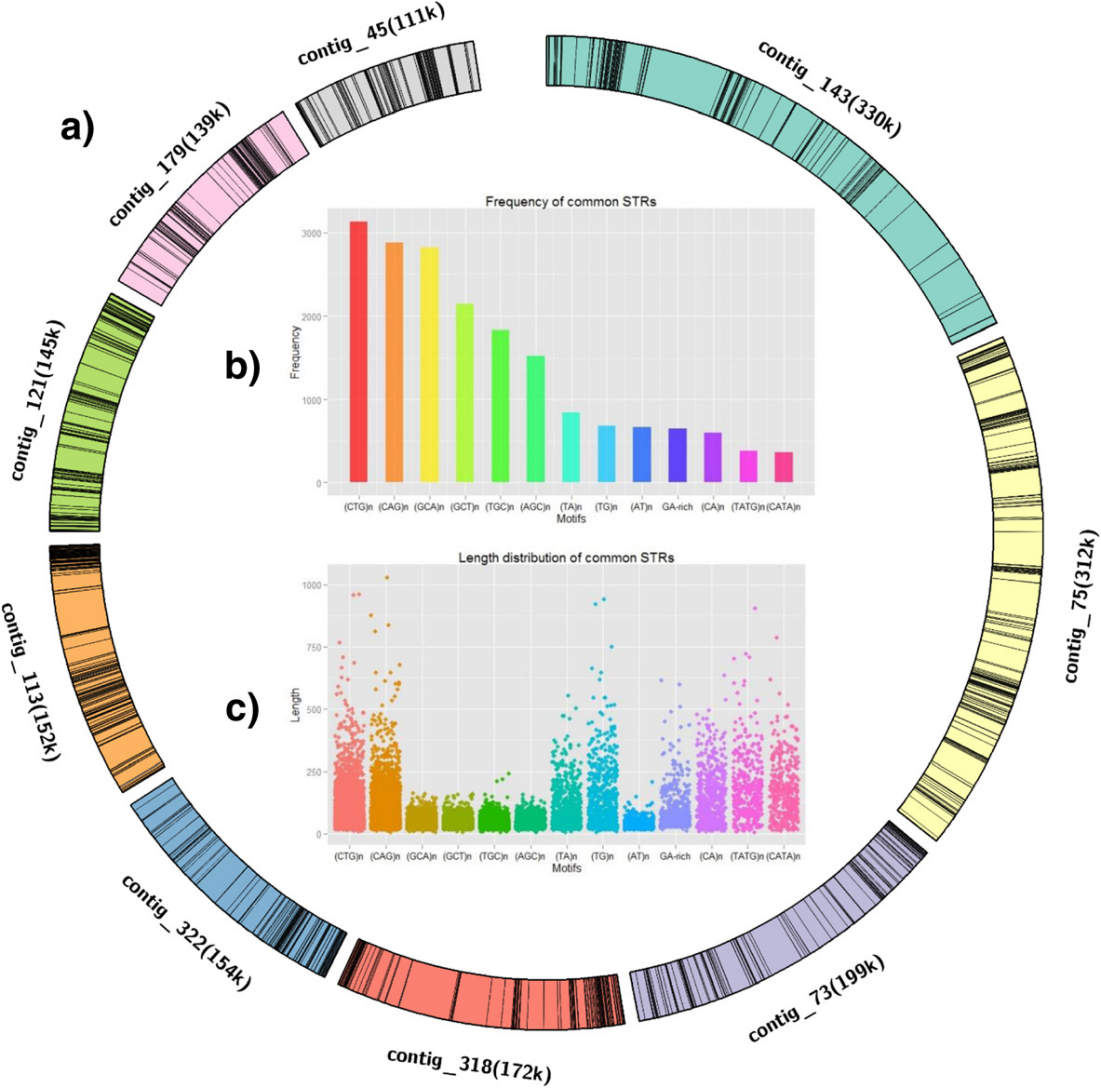
Alternating regions of repeat-rich and repeat-poor sequences and common short tandem repeats (STRs) in the *Cyclospora cayetanensis* genome. A total of 1,700 STRs were detected in the *C. cayetanensis* genome **a** Alternating pattern of repeat-rich and repeat-poor within several large contigs of the *C. cayetanensis* genome. The black bands represent STRs. **b** Distribution of STRs. The most common STRs are the “CAG” motif and its variations, like seen in *Eimeria* spp. **c** Jitter plot showing length distribution of common STRs

The alternation of repeat-rich and repeat-poor regions, which was reported for *Eimeria* spp. [11], was also detected in the *C. cayetanensis* genome (Fig. 1). In addition, the most common short tandem repeats (STRs) are also “CAG” motif and variations of it, as seen in *Eimeria* genomes [11]. There are 87 putative long terminal repeat (LTR) retrotransposons in the *C. cayetanensis* genome (Additional file 4: Figure S2). The length of putative LTRs in *C. cayetanensis* varies from 106 to 996 bp with an average of 337 bp, and the sequence similarity between upstream and downstream LTRs of each retrotransposon varies from 85.0 % to 98.6 %. Cluster analysis showed that they could be divided into 44 types based on sequence identities. Unlike *Eimeria* spp., whose LTR-retrotransposons belong to chromoviruses, neither the chromodomain nor the functional domain of reverse transcriptases was identified in LTR-retrotransposons of *C. cayetanensis*. In a phylogenetic analysis, a representative LTR-retrotransposon sequence from *C. cayetanensis* was placed outside the clade formed by chromoviruses (Additional file 5: Figure S3).

### Gene content

There are 144 predicted tRNA genes in the *C. cayetanensis* genome, which is slightly fewer than 174 in *T. gondii* but much higher than in other apicomplexans. We identified 11 rRNA genes in the draft genome of *C. cayetanensis* (Table 1).The *C. cayetanensis* genome may encode as many as 7457 proteins. Among them, 538 proteins have signal peptides (105 of them target the apicoplast), 1247 had one or more transmembrane regions, and 225 had a GPI-anchor attachment site. These numbers are similar to those in *E. tenella* and *T. gondii* (Table 1).

**Fig. 2:**
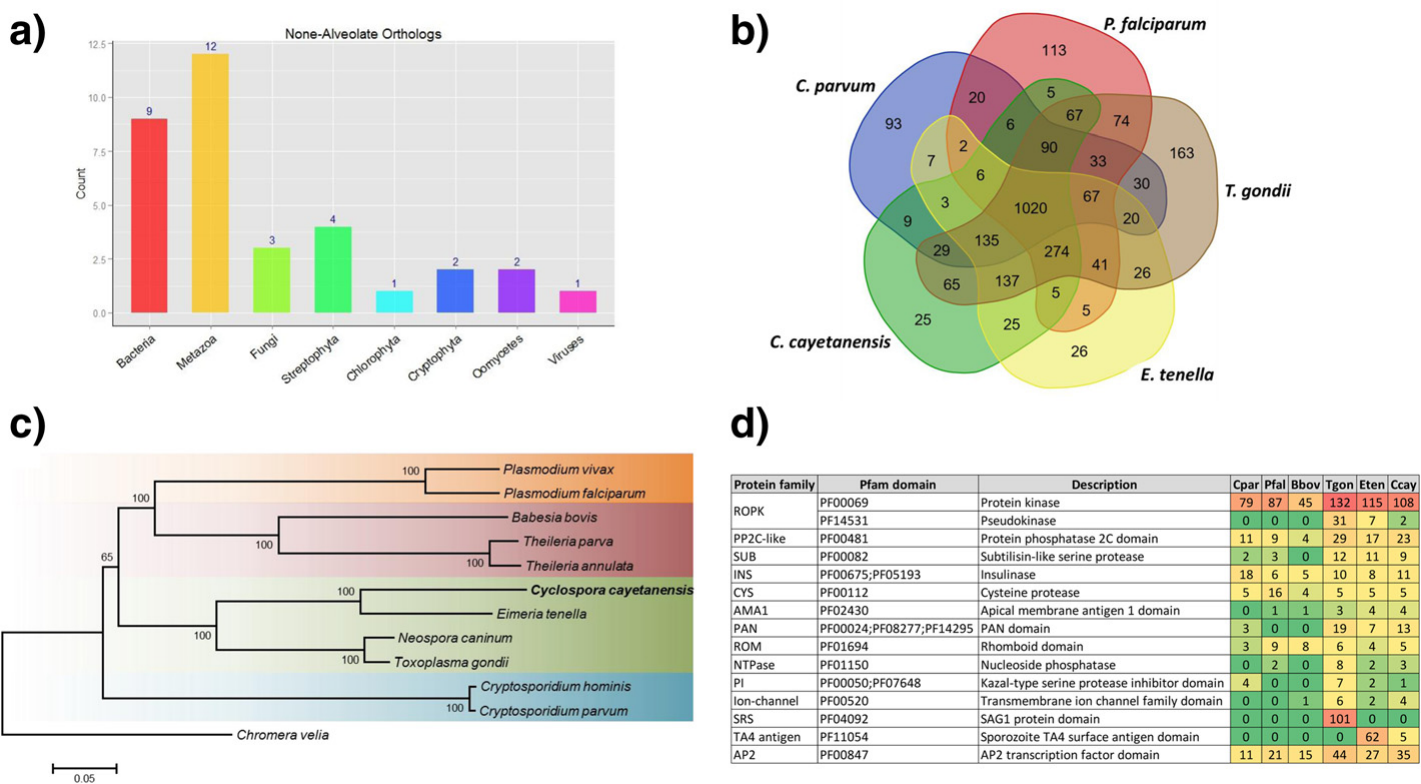
Orthologs in the predicated proteome of *Cyclospora cayetanensis*. **a** In addition to alveolates, a few of the orthologs of *C. cayetanensis* are from other organisms, probably resulted from lateral gene transfers. **b** Functional protein domains shared by apicomplexan parasites *Cryptosporidium parvum*, *Plasmodium falciparum*, *Toxoplasma gondii*, *Eimeria tenella* and *C. cayetanensis*. **c** Phylogenetic relationship of *C. cayetanensis* and other common apicomplexan parasites based on a neighbor-joining analysis of concatenated protein sequences from 100 orthologs; a concatenated sequence from the free-living photosynthetic chromerid, *Chromera velia* was used to root the tree. The maximum composite likelihood method was used in the calculation of genetic distances. Numbers on branches are percent bootstrap values >50 from 1,000 replications. **d** Comparison of major protein families potentially involved in host cell invasion among common apicomplexan parasites. Taxa name abbreviations: *Cryptosporidium parvum* (Cpar); *Plasmodium falciparum* (Pfal); *Babesia bovis* (Bbov); *Toxoplasma gondii* (Tgon); *Eimeria tenella* (Eten); *Cyclospora cayetanensis* (Ccay)

OrthoMCL and BLASTP were used to identify the closest orthologs of the predicted proteins of *C. cayetanensis*. The majority of orthologs were from alveolates (*n* = 6024), but several were from other organisms (*n* = 34) (Fig. 2a). All orthologs of bacterial genes found in *C. cayetanensis* are also present in other apicomplexans, implying a possible origin through lateral gene transfer. By Pfam searching, there is a large group (~1020) of functional domains shared by apicomplexans and a smaller group (~546) by coccidia (Fig. 2b). The heteroxenous *T. gondii* apparently possesses more unique protein domains than the monoxenous *E. tenella* and *C. cayetanensis*. Phylogenetic analysis of 100 orthologous protein sequences confirmed the close relatedness of *C. cayetanensis* to *E. tenella* (Fig. 2c).

### Carbohydrate and energy metabolism

Similar to most other apicomplexans, *C. cayetanensis* depends on carbon metabolism, including glycolysis, tricarboxylic acid (TCA) cycle and pentose phosphate pathways, for energy generation (Table 2, Additional file 6: Table S3). The final product, proton, goes through the electron transport system mediated by a series of membrane-bound mitochondrial enzymes to generate the energy carrier, ATP. The classical NADH dehydrogenase multi-protein complex, complex I, is absent in all apicomplexans, being substituted by an alternative single NADH dehydrogenase [12]. Three other multiple-protein complexes (II-IV) and an ATPase (complex V) are present in *C. cayetanensis*. As a coccidian parasite, *C. cayetanensis* has the capability to store energy in the form of the red algae-like ‘floridean starch’, a variant of amylopectin synthesized by using UDP-Glc (glucose) rather than ADP-Glc used in green algae and land plants [13]. All coccidia have the ability to concatenate UDP-Glc into 1,3-beta-glucans and also likely have a galactose metabolism. *E. tenella* and *C. cayetanensis* have the unique ability to reversely produce mannitol from fructose. A similar pathway may be present in *Cryptosporidium* spp., although it utilizes mannose rather than fructose [12]. The amino and nucleotide sugars, such as UDP-Glc, UDP-GlcNAc (N-acetylglucosamine), and GDP-Man (mannose), are critical resources for the glycosylation of self-generated proteins [12]. All apicomplexans possess this pathway and are able to synthesize these nucleotide sugars. Only coccidia have the enzyme to convert UDP-Glc and UDP-Gal (galactose) in both directions. The reverse conversion between GDP-Man and GDP-Fuc (fucose) is present only in *P. falciparum* and coccidia. *Cryptosporidium* spp. are able to convert UDP-Glc into UDP-GlcA (glucuronate) and then into UDP-Xyl (xylose).

**Table 2:** Comparison of some essential metabolic pathways among common apicomplexan parasites^a^

Category	Metabolic pathway	Cpar	Pfal	Bbov	Tgon	Eten	Ccay
Carbohydrate and energy metabolism	Glycolysis	+	+	+	+	+	+
	Degradation of propionyl-CoA into pyruvate and succinate	-	-	-	+	-	+
	TCA cycle	-	+	+	+	+	+
	Pentose phosphate pathway	-	+	+	+	+	+
	Shikimate biosynthesis	-	+	-	+	+	+
	Folate biosynthesis	-	+	-	+	+	+
	Synthesis of tetrahydrobiopterin/dihydrobiopterin/molybdopterin	-	-	-	+	-	-
	Galactose metabolism	-	-	-	+	+	+
	Synthesis of starch	+	-	-	+	+	+
	Synthesis of trehalose	+	-	+	+	+	+
	Synthesis of 1,3-beta-glucan	-	-	-	+	+	+
	Conversion between UDP-Glc and UDP-Gal	+	-	-	+	+	+
	Conversion between GDP-Man and GDP-Fuc	-	+	-	+	+	+
	Conversion of UDP-Glc to UDP-GlcA then to UDP-Xyl	+	-	-	-	-	-
	Synthesis of mannitol from mannose or fructose	+	-	-	-	+	+
	Fatty acid biosynthesis in cytosol (FAS I)	+	-	-	+	+	+
	Fatty acid biosynthesis in apicoplast (FAS II)	-	+	-	+	+	+
	Fatty acid degradation	-	-	-	+	+	+
	Oxidative phosphorylation (NADH dehydrogenase)	+	+	+	+	+	+
	Oxidative phosphorylation (Complex II)	-	+	+	+	+	+
	Oxidative phosphorylation (Complex III)	-	+	+	+	+	+
	Oxidative phosphorylation (Complex IV)	-	+	+	+	+	+
	F-ATPase	2 subunits	+	+	+	+	+
	V-ATPase	+	+	+	+	+	+
	Glyoxalase metabolism producing D-lactate	-	+	+	+	+	+
	Synthesis of isoprene (MEP/DOXP)	-	+	+	+	+	+
Nucleotide metabolism	Synthesis of purine rings *de novo*	-	-	-	-	-	-
	Synthesis of pyrimidine *de novo*	-	+	+	+	+	+
Amino acid metabolism	Synthesis of alanine from pyruvate	-	-	-	+	+	+
	Synthesis of glutamate from nitrite/nitrate	-	+	+	+	+	+
	Conversion from glutamate to glutamine	+	+	-	+	+	-
	Synthesis of aspartate from oxaloacetate and glutamate	-	+	+	+	+	+
	Conversion from aspartate to asparagine	+	+	-	+	+	+
	Conversion from glutamate to proline	+	-	-	+	+	+
	Synthesis of serine from glycerate/glycerol phosphate	-	-	-	+	+	+
	Conversion from serine to cysteine	-	-	-	+	+	+
	Conversion from serine to glycine	+	+	+	+	+	+
	Recycle homocysteine into methionine	-	+	-	+	-	-
	Synthesis of lysine from aspartate	-	-	-	+	-	-
	Synthesis of threonine from aspartate	-	-	-	+	-	-
	Synthesis of ornithine from arginine	-	+	-	-	-	-
	Synthesis of ornithine from proline	-	+	-	+	+	+
	Synthesis of polyamine from ornithine	-	+	-	-	-	-
	Polyamine pathway backward	+	-	-	+	+	+
	Degradation of leucine to acetyl-CoA	-	-	-	+	-	-
	Degradation of isoleucine/valine	-	-	-	+	+	+
	Aromatic amino acid hydroxylases (AAAH)	-	-	-	+	-	-
Vitamin and others	Synthesis of thiamine (vitamin B1)	-	+	-	-	-	-
	Conversion from thiamine to thiamine pyrophosphate (TPP)	-	+	-	+	-	+
	Synthesis of FMN/FAD from riboflavin	-	+	+	+	+	+
	Synthesis of pyridoxal phosphate (vitamin B6) *de novo*	-	+	-	+	-	-
	Synthesis of NAD(P) + *de novo* from nicotinate/nicotinamide	-	+	-	+	+	+
	Synthesis of pantothenate from valine	-	-	-	+	+	+
	Synthesis of CoA from pantothenate	+	+	+	+	+	+
	Synthesis of lipoic acid *de novo* in apicoplast	-	+	-	+	+	+
	Salvage lipoic acid in mitochondria	-	+	+	+	-	+
	Synthesis of porphyrin/cytochrome proteins	-	+	-	+	+	+

^a^Plus symbol denotes that the essential enzymes for pathways were identified, whereas minus symbol denotes that the essential enzymes for pathways were absent. Only 2 subunits of the F-type ATPase are present in *Cryptosporidium parvum*. Abbreviation: *Cryptosporidium parvum* (Cpar); *Plasmodium falciparum* (Pfal); *Babesia bovis* (Bbov); *Toxoplasma gondii* (Tgon); *Eimeria tenella* (Eten); *Cyclospora cayetanensis* (Ccay)

Within the pyruvate metabolism, *C. cayetanensis* and *E. tenella* possess neither the phosphoenolpyruvate (PEP) carboxylase utilized by *P. falciparum* and *Cryptosporidium* spp. nor the pyruvate carboxylase present in *T. gondii* [12]. However, a PEP carboxykinase is present in *C. cayetanensis, E. tenella* and other apicomplexans except *Cryptosporidium* spp., allowing them to continuously produce oxaloacetate to supplement the TCA cycle. In *P. falciparum* and *T. gondii*, the glycolysis and TCA cycle are disconnected due to the fact that the pyruvate dehydrogenase complex is localized in apicoplasts rather than mitochondria [14]. In addition to all enzymes needed for the TCA cycle in mitochondria, the aconitase dually targeting the mitochondria and apicoplast and an isoenzyme of isocitrate dehydrogenase (ICDH1) targeting the apicoplast are present in *T. gondii,* suggesting that a partial TCA cycle exists in its apicoplast [12, 15]. The genes encoding two aconitases, the ortholog of ICDH1, and isoforms of citrate synthases were detected in nuclear genomes of *C. cayetanensis* and *E. tenella*. Thus, *C. cayetanensis* probably also possesses a partial TCA pathway in its apicoplast.

Like most other apicomplexan parasites, *C. cayetanensis* probably uses the pentose phosphate pathway to produce *de novo* phosphoribosyl pyrophosphate (PRPP), which is involved in pyrimidine biosynthesis. A ribokinase is present only in *T. gondii*, *E. tenella* and *C. cayetanensis*, suggesting that only coccidia are able to salvage ribose from the host in addition to synthesizing it *de novo*. Compared to *P. falciparum* and *T. gondii*, the deoxyribose phosphate aldolase for deoxyribose catalysis is absent in both *E. tenella* and *C. cayetanensis*. Another important intermediate within the pentose phosphate pathway, erythrose-4-phosphate, is the substrate in biosynthesis of shikimate as well as folate, which is eventually converted into tetrahydrofolate (THF) and methylene-THF. These two folates are essential for nucleotide conversion and amino acid conversion, respectively. In addition to the *de novo* synthesis of folates, apicomplexan parasites can transport folic acid from the extracellular environment using specific cytosol membrane transporter proteins (Table 3). Furthermore, *T. gondii* possesses two extended sub-pathways for folate metabolism: 1) the biosynthesis of dihydrobiopterin and tetrahydrobiopterin, which can provide hydroxyl for converting phenylalanine to tyrosine; and 2) the biosynthesis of molybdopterin, the cofactor for sulfite oxidation [12]. None of these enzymes or proteins were identified in *C. cayetanensis*.

**Table 3:**
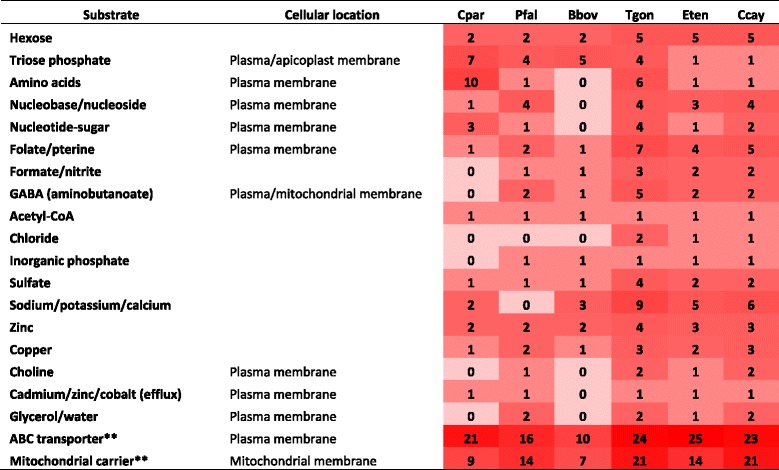
Putative transporters in common apicomplexan parasites*

*The detection of putative transporter proteins was based on Pfam search. Abbreviation: *Cryptosporidium parvum* (Cpar); *Plasmodium falciparum* (Pfal); *Babesia bovis* (Bbov); *Toxoplasma gondii* (Tgon); *Eimeria tenella* (Eten); *Cyclospora cayetanensis* (Ccay)

**ABC transporter and mitochondrial carrier have a broad range of substrates

Fatty acid biosynthesis in apicomplexans is thought to occur in the apicoplast through type II fatty acid synthases encoded in the nuclear genome [14]. Some apicomplexans also possess the prokaryotic type I fatty acid synthase in the cytosol to elongate short-chain fatty acids salvaged from the host [14]. The genes coding both types of fatty acid synthases are present in the *C. cayetanensis* genome, similar to *E. tenella* and *T. gondii* (Table 2, Additional file 6: Table S3). Most apicomplexans synthesize isoprenoids in the apicoplast through a bacteria-type DOXP pathway utilizing phosphoenol pyruvate and dihydroxyacetone phosphate [14]. The complete set of enzymes involved in isoprenoid biosynthesis including the apicoplast glyceraldehyde-3-phosphate dehydrogenase isoenzyme characterized in *T. gondii* [16] was detected in *C. cayetanensis*.

### Amino acids metabolism

Similar to *T. gondii* and *E. tenella*, *C. cayetanensis* can synthesize alanine from pyruvate while other apicomplexans have to salvage it from the host (Table 2, Additional file 6: Table S3). Except for *Cryptosporidium* spp., all apicomplexans including *C. cayetanensis* can utilize nitrite or nitrate transported from the host to synthesize glutamate, which can be converted into glutamine through glutamine synthetase in coccidia and *P. falciparum*. Except for *Cryptosporidium* spp., all apicomplexans can reversely convert oxaloacetate and glutamate to aspartate. Only coccidia have the ability to generate proline from glutamate as in humans, the enzymes for producing serine *de novo* from glycerate or glycerol phosphate, and the inter-converting serine into cysteine as in humans and animals.

Among apicomplexans, only *T. gondii* possesses the capacity of biosynthesis of lysine and threonine from aspartate, whilst there is only a putative threonine synthase in *C. cayetanensis*. Another important amino acid in apicomplexans, methionine, is probably salvaged from the host, and can be converted to homocysteine, the substrate for the biosynthesis of cysteine. In *P. falciparum* and *T. gondii,* homocysteine can be potentially recycled into methionine [12]. Due to the lack of an arginase, no coccidia can synthesize ornithine, the substrate for the biosynthesis of polyamines from arginine, as in *P. falciparum*. However, coccidia have the capability to convert proline into ornithine. Only *P. falciparum* can synthesize polyamines, spermidine and spermine through putrescine (http://mpmp.huji.ac.il). However, all coccidia can probably synthesize putrescine reversely from spermine salvaged from the host.

No apicomplexans are able to synthesize aromatic amino acids *de novo*; they have to salvage them from the host through an amino acid transporter embedded in the plasma membrane [12]. *C. cayetanensis* and *E. tenella,* however, each has only one amino acid transporter, compared with 10 in *Cryptosporidium* spp. and 6 in *T. gondii* (Table 3). Some of the >20 ABC transporters present in each genome could be responsible for the salvage of some aromatic amino acids. Within the phenylalanine and tyrosine catabolism pathway, there are two aromatic amino acid hydroxylases in *T. gondii,* catalyzing the hydroxylation of phenylalanine to synthesize tyrosine and L-DOPA [12]. The genes encoding these enzymes were not detected in *C. cayetanensis* and other apicomplexans. For the catabolism of branched chain amino acids, only *T. gondii* potentially has the ability to generate acetyl-CoA through the degradation of leucine. Compared to *P. falciparum,* which possesses only the early steps of the pathway, coccidia can degrade isoleucine and valine to generate propionyl-CoA and (R)-methyl-malonyl-CoA, respectively, supplementing intermediates for the TCA cycle [12]. *T. gondii* and *C. cayetanensis* have the full set of enzymes for the degradation of propionyl-CoA, generating pyruvate and succinate. In addition, *T. gondii* has a pyruvate carboxylase, catalyzing pyruvate to oxaloacetate to make the methyl-citrate cycle a full pathway, similar to bacteria and fungi [12].

### Nucleotide metabolism

No apicomplexans have the ability to synthesize purine rings *de novo* and have to salvage them from the host (Table 2, Additional file 6: Table S3). There are four homologous genes encoding nucleoside transporters in *C. cayetanensis* (Table 3). In addition, the presence of an adenosine kinase (AdK) indicates that adenosine may be the major purine utilized by *C. cayetanensis*, in contrast to the AMP used by *P. falciparum* [17, 18]. Like most other apicomplexans except *Cryptosporidium* spp., *C. cayetanensis* possesses all the enzymes for synthesizing pyrimidine *de novo* from aspartate and glutamine, except for the orotate phosphoribosyl transferase that catalyzes the phosphorylation of orotate using PRPP. In line with a parasitic life style, coccidian parasites have a salvage pathway for pyrimidine in addition to its *de novo* biosynthesis.

### Coenzymes, vitamins and other metabolism

Similar to *P. falciparum*, *T. gondii* and *E. tenella, C. cayetanensis* possesses almost all enzymes needed to synthesize the coenzymes NAD^+^ and NADP^+^ from nicotinate (Table 2, Additional file 6: Table S3). Coccidia can synthesize acyl-chain carrier coenzyme A (CoA) *de novo* from valine, but other apicomplexans have to salvage pantothenate from the host and convert it into CoA. In apicomplexans, only *P. falciparum* possesses the enzymes synthesizing thiamine from intermediates, whereas other apicomplexan parasites have to salvage it from the host. A single enzyme reaction that catalyzes pyro-phosphorylation of thiamine producing thiamine pyrophosphate, the active form of vitamin B1, is present in *P. falciparum*, *T. gondii* and *C. cayetanensis*. The absence of pyridoxal 5-phosphate (PLP) synthase in *C. cayetanensis* and *E. tenella* suggests that these parasites may have lost the ability to synthesize PLP, a component of vitamin B6, *de novo* from glutamine. However, the salvage pathways through the phosphorylation of pyridoxal or oxidation of pyridoxine/pyridoxamine phosphate are present in *C. cayetanensis* [12]. It has been shown that lipoic acid (LA), the critical cofactor for some dehydrogenase complexes, can be synthesized *de novo* in the apicoplast, or salvaged from the host and utilized in the mitochondrion in *T. gondii* [12, 19]. The catalytic enzymes involved in the LA metabolism were all detected in *C. cayetanensis. E. tenella* lacks the enzymes used in mitochondria, but possesses dehydrogenase complexes similar to *T. gondii* and *C. cayetanensis,* suggesting that this inferred gene loss may not be true.

### GPI-anchor, N-glycan, and mucin-type O-glycan biosynthesis

**Fig. 3:**
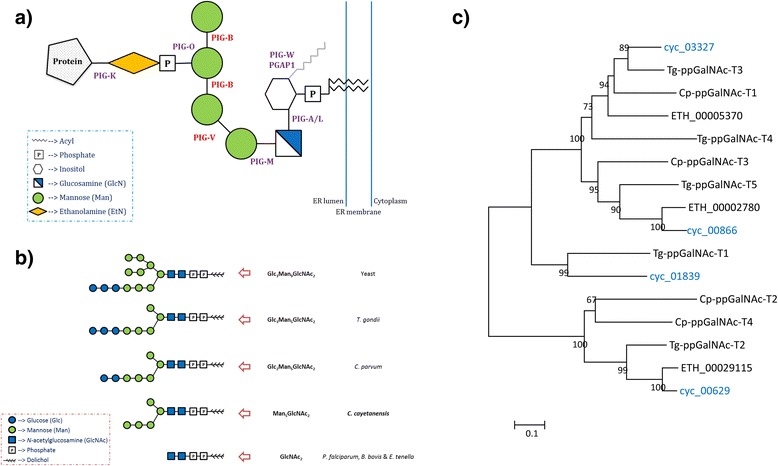
Post-translational modifications of proteins in *Cyclospora cayetanensis* and other apicomplexans. **a** Core structure of GPI-anchor precursor and critical enzymes involved in its biosynthesis. Genes encoding PIG-V and PIG-B (shown in red) are absent in the *C. cayetanensis* genome. **b** Structure of hypothetical *N*-glycan precursors in different apicomplexans. Due to the secondary loss of ALG-encoding genes, the precursors of *N*-glycan are divergent in apicomplexans from 10 sugars in *Toxoplasma gondii* to 2 sugars in *Plasmodium falciparum* and *Eimeria tenella. C. cayetanensis* possesses a 7 sugar precursor which is just enough for glycosylation but not sufficient for N-glycan-dependent quality control of protein folding. **c** Phylogenetic relationship of ppGalNAc-Ts, the critical enzyme in mucin-type O-glycan biosynthesis, from *Cryptosporidium parvum* (Cp), *T. gondii* (Tg), *E. tenella* (ETH), and *C. cayetanensis* (cyc) based on a neighbor-joining analysis using genetic distances calculated with the maximum composite likelihood method. Numbers on branches are percent bootstrap values >50 from 1,000 replications. *C. cayetanensis* has ppGalNAc-Ts similar to other coccidia, especially *E. tenella*

Most surface antigens of apicomplexans involved in host cell recognition, interaction or adhesion use a glycosylphosphatidylinositol (GPI) anchor for attachment to the plasma membrane, such as SRS (SAG1-related sequences) proteins of *T. gondii* and TA4-type surface antigens of *E. tenella* [11]. Two essential mannosyltransferases in the biosynthesis of the GPI-anchor, PIG-V and PIG-B, were not identified in *C. cayetanensis* and *E. tenella* (Fig. 3a). In addition, the modification of the inositol residue during the construction of the GPI-anchor in the ER lumen is different among apicomplexans. Coccidia can both acylate (PIG-W) and de-acylate (PGAP1) inositol, while *P. falciparum* and *Babesia bovis* can only acylate, and *Cryptosporidium* spp. have lost both capacities.

N-linked glycans, oligosaccharides attached to the asparagine (Asn) residue in a tripeptide sequence of Asn-X-Ser/Thr (where X is any amino acid except Pro) of proteins, are very common in eukaryotes [20]. Based on the presence and absence of critical enzymes involved in the biosynthesis of N-glycan precursors, we have predicted putative final N-glycan precursor structures in different apicomplexans (Fig. 3b). Compared to *T. gondii* and *Cryptosporidium* spp., *C. cayetanensis* does not add any glucose onto the core structure of the N-glycan precursor. In contrast, the enzymes that catalyze the addition of oligosaccharides onto the *N*-acetylglucosamines (GlcNAc) during N-glycan biosynthesis are absent in *E. tenella, P. falciparum* and *B. bovis*. During the trimming process, the glucosidase needed for removing the external glucose is absent in *C. cayetanensis* while another glucosidase involved in removing the remaining two glucoses is present.

Mucin type O-glycosylation is another common post-translational modification of proteins especially those from the secretory organelles of apicomplexans [21]. The enzymes catalyzing the biosynthesis of O-glycans have not been characterized for apicomplexans, except for the initial enzyme, UDP-GalNAc: polypeptide *N*-acetylgalactosaminyltransferase (ppGalNAc-T), which transfers GalNAc from UDP-GalNAc to the hydroxyl group of specific serine or threonine residues in proteins [21, 22]. Four putative ppGalNAc-Ts from distinct families were identified in *C. cayetanensis* (Fig. 3c).

### Adhesins, surface antigens and glideosome

By function, the super families of secreted proteins in the apical complex can be separated into three groups: i) adhesins involved in binding and interaction with host cells during the initial invasion, ii) secreted or membrane-associated peptidases involved in processing rhoptry and micronemal proteins of parasites and degrading proteins of the host, and iii) secreted signaling proteins such as protein phosphatases and kinases, which are injected across the plasma membranes into the host cell cytoplasm or nucleus, modulating host cell signaling pathways or immune responses to promote the survival of parasites [23]. Some of the major host cell invasion-related protein families were compared among common apicomplexans, which has shown some diversity in major surface antigens and protein kinases (Fig. 2d).

Based on the type of adhesins shared among parasites, *C. cayetanensis* and *E. tenella* probably have an adhesive system very similar to that of *T. gondii* (Additional file 7: Table S4). Some major differences, however, were seen in the type of major surface antigens among coccidia. There are a large number of surface antigens called SRS proteins on the surface of *T. gondii,* approximately doubling the number in *Neospora caninum,* a close relative of *T. gondii* [24]. These highly expressed surface proteins are thought to be involved in the attachment of parasites to host cells and potentially to be responsible for the broad host range of *T. gondii* [25]. In *E. tenella*, the principal surface antigen genes (89 genes in three subfamilies) are arrayed in four gene clusters. Their products, TA4-type surface antigens containing signal peptides and GPI-anchor sites, are thought to interact with host cell prior to invasion [11]. We did not find any cluster of genes encoding proteins with signal peptides and GPI-anchor sites in the *C. cayetanensis* genome. Only four putative TA4-type surface antigens, which are more similar to the subfamily SagA of *Eimeria* spp., were identified in *C. cayetanensis* and one of them has both a signal peptide and a GPI-anchor. The cysteine-rich secretory protein family (CAP), which TA4 surface antigens probably derived from, was also detected in the *C. cayetanensis* genome. By paralog analysis using OrthoMCL, a large group comprised of 31 genes annotated as hypothetical proteins were found in the *C. cayetanensis* genome. Some (*n* = 11/31) have cytosol membrane-related or periplasmic substrate binding-related functions (Additional file 8: Table S5). One of these paralogs has some sequence similarity to erythrocyte membrane protein 1 (PfEMP1), which is involved in erythrocyte invasion by *P. falciparum* [26]. The length of these paralogous genes varies from 243 bp to 3627 bp, compared with ~700-800 bp in the TA4 genes of *E. tenella*.

In *T. gondii* and *P. falciparum*, the power source of gliding and invasion comes from a motor complex consisting of myosin, gliding associated protein (GAP) and some other proteins [27]. The homologs of all of these proteins were found in *C. cayetanensis* and *E. tenella* suggesting that the motor structure may be conserved within all apicomplexans (Additional file 7: Table S4). After the initial attachment to host cells, *T. gondii* forms a moving junction, the AMA1-RON complex, to anchor the parasite to the host cell cytoskeleton [27, 28]. *C. cayetanensis* and *E. tenella* possess homologs for these proteins, suggesting that their host cell attachment system is similar to that in *T. gondii*.

### Secreted proteases and protein kinases

Proteases and peptidases produced by the apical complex are thought to either modify other secreted apical complex-related proteins that function in the extracellular environment or degrade host proteins after crossing the plasma membrane. One serine protease, subtilisin in *T. gondii* (TgSUB1), is required for the processing of microneme proteins, affecting the efficiency of adhesion of tachyzoites [29]. Although the ortholog of TgSUB1 was not found in *C. cayetanensis* and *E. tenella*, the ortholog of another rhoptry subtilisin-like protease with specificity similar to the ROP1 mutarase [30], TgSUB2, was found in these two parasites (Additional file 9: Table S6). Thus far, two cysteine endoproteases, cathepsins B (TgCPB) and L (TgCPL), and three cysteine exoproteases, cathepsins C1 to C3 (TgCPC1, TgCPC2 and TgCPC3), have been characterized in *T. gondii* and are known to play essential roles in the growth and intracellular survival of parasites [31]. Except for CPC3, which is present in *E. tenella*, *C. cayetanensis* has four members of these two types of proteases. Even though the substrate for metalloproteinases, named toxolysins, is unclear, the presence of a rhoptry pro-domain cleavage site within toxolysin-1 (TLN1) suggests that toxolysins are probably protein maturases [32]. Rhomboid proteases (ROMs) are a family of intramembrane serine proteases in all kingdoms of life, and were shown to be responsible for the cleavage of secreted adhesive proteins in apicomplexans. Among them, TgROM4 functions as a micronemal protease and is essential for host cell invasion of *T. gondii* [33, 34], whereas TgROM2 and TgROM5 are thought to cleave the transmembrane domains of some MICs that are involved in gliding and invasion [35]. The homolog of TgROM3 was not identified in *C. cayetanensis,* whereas homologs of TgROM2 and TgROM6 were not identified in *E. tenella* (Additional file 9: Table S6).

**Fig. 4:**
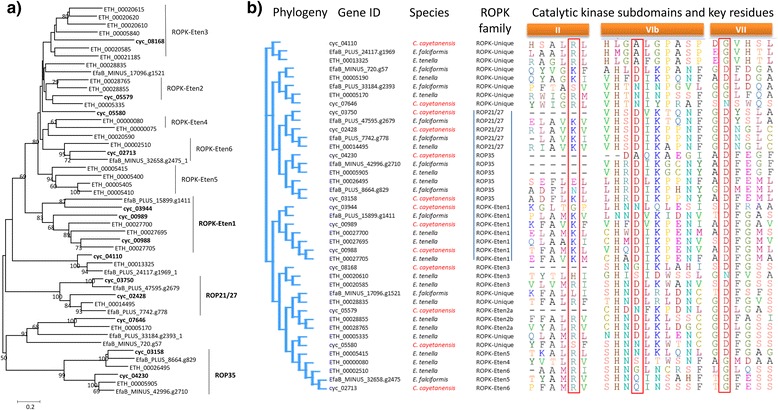
Phylogeny of putative rhoptry protein kinase (ROPK) families and conserved catalytic domains in *Eimeria tenella*, *E. falciformis* and *Cyclospora cayetanensis*. **a** Phylogenetic relationship among ROPKs. The neighbor-joining phylogenetic tree was constructed based on genetic distances calculated using the maximum composite likelihood method. Numbers on branches are percent bootstrap values >50 from 1,000 replications. The ROPKs from *C. cayetanensis* are shown in bold. **b** Catalytic kinase subdomains and key residues of ROPK subfamilies. The catalytic lysine in subdomain II and aspartic acids in subdomain VIb and VII are considered the key residues in active protein kinases. Their presence along with five other residues in different subdomains suggests that sub-families ROP21/27, ROP35, and ROPK-Eten1 are active protein kinases; the remaining sub-families are either inactive or potentially non-canonical rhoptry kinases. In *C. cayetanensis*, at least four ROPKs, including cyc_02428 from the ROP21/27 subfamily, cyc_03158 from the ROP35 subfamily, cyc_00989 and cyc_00988 from the ROPK-Eten1 subfamily, are active protein kinases

Apicomplexans have the ability to modulate host cell metabolism, especially the signaling pathways to allow them to evade the host immune system. *T. gondii* possesses a special secretory protein phosphatase 2C (PP2C-hn) secreted by the rhoptry and delivered into host cell nuclei during invasion [36]. There are no orthologs of PP2C-hn in *C. cayetanensis* and *E. tenella* (Additional file 10: Table S7). Some PP2C-like secretory phosphatases were identified in *C. cayetanensis* and *E. tenella*, but their numbers are smaller than seen in *T. gondii*. In addition, rhoptries also release a range of protein kinases (ROPK) to modulate host cell functions. The best known is TgROP18, which phosphorylates and inactivates host immunity-related GTPases [37, 38]. ROP5, ROP16 and ROP38 are also implicated in the modulation of host immune responses or signaling pathways. These ROPKs do not have any orthologs within *C. cayetanensis* and *E. tenella. E. tenella* has a smaller number of ROPKs and several *E. tenella-*specific groups of ROPKs [11]. We identified 13 putative ROPK-encoding genes in the *C. cayetanensis* genome, significantly smaller than the number in the *E. tenella* genome but similar to that in *E. falciformis* [39] (Table 4). The putative ROPKs of *C. cayetanensis* are ROP21/27/35-like and *E. tenella*-specific ROPKs (Fig. 4a). Among them, ROP21/27, ROP35 and ROPK-Eten1 subfamilies have conserved catalytic residues of ROPKs [40] (Fig. 4b), suggesting that these coccidia likely have some capacity to modify host signaling pathways. Overall, the number of known secretory ROPKs in *C. cayetanensis* is significantly reduced, and two of them, ROPK-Eten4 and ROPK-Eten5, appear to be orthologs of *E. tenella* ROPKs.

**Table 4:** Predicted rhoptry protein kinases (ROPKs) in *Cyclospora cayetanensis* using HMM profiles search and their orthologs in other coccidia

Gene ID	Best hit HMM family	E-value	Score	General PK score	*E. tenella*	*E. falciformis*	*T. gondii*
cyc_02428	ROP21/27	1.4E-104	348.6	94.4	ETH_00014495	EfaB_PLUS_7742.g778	TGME49_263220
cyc_03750	ROP21/27	3.1E-100	334.4	82.9		EfaB_PLUS_47595.g2679	TGME49_313330
cyc_04230	ROP35	1.6E-39	134.9	40.3	ETH_00005905	EfaB_MINUS_42996.g2710	
cyc_03158	ROP35	4.3E-83	277.5	89.3	ETH_00026495	EfaB_PLUS_8664.g829	TGME49_304740
cyc_00988	ROPK-Eten1	3.0E-108	361.3	75.0	ETH_00027705	EfaB_PLUS_15899.g1411	
cyc_00989	ROPK-Eten1	2.6E-77	259.6	79.2	ETH_00027695		
cyc_03944	ROPK-Eten1	4.0E-29	100.8	57.2	ETH_00027700		
cyc_05579	ROPK-Eten2a	3.9E-60	202.5	78.4	ETH_00028765		
	ROPK-Eten2b				ETH_00028855		
cyc_08168	ROPK-Eten3	1.1E-35	122.2	40.4	ETH_00020585		
	ROPK-Eten3				ETH_00020615 ETH_00020590		
					ETH_00020610 ETH_00005840		
					ETH_00021185 ETH_00020620		
	ROPK-Eten4				ETH_00000075 ETH_00000080		
	ROPK-Eten5				ETH_00005415 ETH_00005400		
					ETH_00005405 ETH_00005410		
cyc_02713	ROPK-Eten6	1.6E-66	223.3	64.0	ETH_00002510	EfaB_MINUS_32658.g2475	
cyc_05580	ROPK-Unique	1.3E-71	240.3	78.2	ETH_00028835	EfaB_MINUS_17096.g1521	
cyc_04110	ROPK-Unique	4.1E-56	189.4	28.0	ETH_00013325	EfaB_PLUS_24117.g1969	
cyc_07646	ROPK-Unique	1.1E-48	165.0	41.4	ETH_00005170	EfaB_PLUS_33184.g2393	
	ROPK-Unique				ETH_00005335

In *T. gondii*, there are two potent nucleoside triphosphate hydrolases, NTPase I and NTPase II, which are localized in dense granules and secreted into the PV, affecting host signaling pathways during invasion [23]. Both of them are absent in *C. cayetanensis* and *E. tenella*. Protease inhibitors in *T. gondii*, TgPI-1 and TgPI-2, are dense granule proteins secreted into the PV to potentially inhibit trypsin, chymotrypsin, neutrophil and pancreatic elastases, protecting the parasite from host immune responses [41]. The lack of these catalytic proteins with functional domains, such as Kazal in TgPI proteins, may be partially responsible for the strict tissue tropism in *C. cayetanensis* and *E. tenella*. There is also a large group of secretory proteins stored in dense granules called GRAs in *T. gondii*, which have no identifiable Pfam domains but are essential for invasion and egress. One of them, GRA15, like some rhoptry proteins is delivered across the PV membrane to modulate host cell signal pathways. Two others, GRA16 and GRA24, have been demonstrated to target the host cell nucleus, affecting host gene expression [9]. Except for GRA9/10/11/12, there are no homologs of these proteins in *C. cayetanensis* and *E. tenella*.

### Transcription factors

Apicomplexans have a major transcription factor family called the apicomplexan AP2 family of proteins (ApiAP2), with some similarities to the plant AP2 [42, 43]. In *T. gondii*, TgAP2s regulate stage-specific expression of genes. At least 35 ApiAP2 domain-containing proteins are encoded by *C. cayetanensis*. This is less than the 44 ApiAP2 proteins in *T. gondii*, but more than the 27 in *E. tenella*.

## Discussion

Comparative genomic analysis indicates that *C. cayetanensis* shares some of the genomic features and metabolic capabilities of coccidia such as *T. gondii* and *E. tenella*. Compared with the metabolism in *T. gondii*, *C. cayetanensis* and *E. tenella* primarily lack *de novo* biosynthesis of certain amino acids and the ability to salvage amino acids directly from the host is significantly reduced. Differences in the degradation and hydroxylation pathways of some amino acids were also observed among the coccidian parasites examined. It appears amino acid metabolism evolves more rapidly in coccidia than other metabolic pathways. It is possible the lack of these amino acid metabolic pathways has reduced the target tissue range in *C. cayetanensis* and *E. tenella*. The only unique metabolic pathway present in *C. cayetanensis* and *E. tenella* but absent in *T. gondii* is the synthesis of mannitol from fructose catalyzed by a single enzyme. The fungi-like mannitol cycle metabolism (fructose-mannitol-mannitol phosphate-fructose phosphate-fructose) was known to be present in *E. tenella* [44]. Mannitol is accumulated as an energy reserve during oocyst formation in the host and utilized for sporulation outside of the host [45]. It is probably vital to the sporulation and survival of *C. cayetanensis* oocysts. A clear difference between *C. cayetanensis* and *E. tenella* is the ability of the former to degrade propionyl-CoA to produce pyruvate and succinate, probably as supplements for the TCA cycle, suggesting that *C. cayetanensis* probably has more carbon sources for mitochondrial metabolic activities than *E. tenella*.

The mechanisms involved in post-translational modifications of proteins may be different between *C. cayetanensis* and *Eimeria* spp. Because of the secondary loss of ALG enzymes, the length of N-glycan precursors is divergent among apicomplexans, from 10 sugars in *T. gondii* to 2 in *E. tenella* and none in *Theileria* spp. [46]. The N-glycan precursor in *C. cayetanensis* possesses 7 sugars, Man_5_GlcNAc_2_. Similarly, a paucity of enzymes involved in the biosynthesis of GPI-anchor was detected in *C. cayetanensis* and *E. tenella*. Both *C. cayetanensis* and *E. tenella* lack mannosyltransferases PIG-V and PIG-B. In addition, phosphomannomutase and dolichol-phosphate-mannose (Dol-P-Man) synthase, the enzymes involved in generation of Dol-P-Man, which is the substrate for N-glycan and GPI-anchor synthesis, are absent in *Eimeria* spp. [39] but are present in *C. cayetanensis*. Thus, there are substantial differences in both GPI anchor biosynthesis and N-glycosylation between *C. cayetanensis* and *Eimeria* spp.

The similar repertoire of host cell invasion-related proteins possessed by all coccidian parasites suggests that *C. cayetanensis* has host cell receptors and invasion process similar to that of *T. gondii* and *E. tenella*: gliding powered by an actin motor before invasion, interactions with the host cell through surface antigens, forming a moving junction with a series of secreted proteins to initiate invasion, forming a PV structure inside the host cell, and secreting various protein kinases, protein phosphatases, and other catalytic proteins to modify the host metabolic pathways for the evasion of host immune responses. The amplification and diversification of surface and secreted proteins are probable determinants for distinct transmission, host range and pathogenicity of various coccidia [47]. Compared with *T. gondii*, the dramatic reduction in protein kinases and phosphatases suggests that *C. cayetanensis* and *E. tenella* have only limited capacity to regulate host cell signaling pathways and gene expression, or use a divergent system to do so. These genomic characteristics of *E. tenella* and *C. cayetanensis* are probably responsible for their exclusive enteric life cycle. Between *C. cayetanensis* and *E. tenella*, the former has a further reduction in the number and type of ROPKs.

Surface antigens SRS (SAG1-related sequences) are involved in initial interaction with host cells in *T. gondii* invasion [48]. *N. caninum* possesses the same type of SRS proteins seen in *T. gondii*, but has a significant increase in their number [24]. Neither *C. cayetanensis* nor *E. tenella* has this family of surface proteins. In contrast, *Eimeria* spp. have the unique TA4-type surface antigens and show divergence in their compositions among species [11]. In the *C. cayetanensis* genome, we detected several TA4-type surface antigen coding genes in different genomic regions. Thus, surface antigens are probably the most rapidly evolved proteins in coccidia and are likely determinants for host specificity. We assume that *C. cayetanensis* possesses its own unique surface antigens. The paralogous genes we identified in this study encode mostly hypothetical proteins, one of which has sequence homology to the PfEMP1 of *P. falciparum*. They probably represent the surface antigens of *C. cayetanensis*, as many of these proteins are predicted to have membrane-related or periplasmic substrate binding-related functions. Further studies on the expression, localization and neutralization ability of these proteins are needed to confirm their surface antigen nature in *C. cayetanensis.*

## Conclusions

Through whole genome sequencing and comparative genomic analysis, we have shown that *C. cayetanensis* probably possesses a classical coccidian metabolism and has a host cell invasion system very similar to *Eimeria* spp. and *T. gondii*. The amino acid metabolism and post-translation modifications of proteins are probably the most rapidly evolved metabolic pathways among coccidia. Compared with the heteroxenous *T. gondii*, the monoxenous *C. cayetanensis* and *Eimeria* spp. appear to have very limited abilities or use different mechanisms to modulate host nuclear activities and signaling pathways during invasion. The dominant surface antigens seen in other coccidia are not present or are significantly reduced in number in *C. cayetanensis* and the presence of divergent surface proteins among coccidia suggests that these proteins are likely determinants of host specificity. These observations, however, are based on results of comparative genomic analyses and need to be validated by functional studies. Overall, the availability of whole genome sequence data has significantly improved our understanding of the biology of *C. cayetanensis* and may facilitate the development of molecular diagnostic tools for traceback studies of foodborne cyclosporiasis outbreaks.

## Methods

### Sample collection and DNA preparation

The *C. cayetanensis* specimen sequenced in this study was collected in July 2011 from a patient with severe diarrhea in Kaifeng, Henan, China, where cyclosporiasis is endemic and *C. cayetanensis* isolates were characterized morphologically and by sequence analysis of the SSU rRNA gene [49]. It was diagnosed in this study through acid-fast microscopy and confirmed as *C. cayetanensis* by ultraviolet epifluorescence microscopy and PCR analysis of a ~680-bp fragment of the SSU rRNA gene [10]. DNA sequences obtained from three PCR products were identical to each other and had only an A to G substitution at nucleotide 72 of the GenBank reference sequence AF111183. *C. cayetanensis* oocysts were purified from the specimen using sucrose and cesium chloride gradients [50] and further purified twice by flow cytometry sorting on a FACSAria III (BD Biosciences, San Jose, CA). A gate on forward and side scatter profiles, a gate on autofluorescence, and detectors and filters appropriate for propidium iodide (PI) and fluorescein isothiocyanate were used in sorting. The oocysts in suspension were stained with 1.5 micrograms/ml of PI and a 488 nm laser was used for excitation. Total genomic DNA was extracted from 6 × 10^6^ oocysts using a QIAamp®DNA Mini Kit (Qiagen Sciences, Maryland, 20874, USA), after the oocysts were subjected to five freeze-thaw cycles and overnight digestion with proteinase K. About 100 ng of extracted DNA was amplified using REPLI-g Midi Kit (Qiagen GmbH, Hilden, Germany) according to the manufacturer-recommended procedure.

### Library construction, sequencing, and assembly

The *C. cayetanensis* isolate was sequenced on a Roche 454 GS-FLX Titanium System (Roche, Branford, CT) using the standard Roche library protocol, and on an Illumina Genome Analyzer IIx and a Hiseq 2500 (Illumina, San Diego, CA) using the Illumina TruSeq (v3) library protocol. For Roche 454 sequencing, sequence reads of approximately 400 bp were generated in one run and 450 bp in another, whereas in Illumina sequencing, 100 × 100 bp paired-end reads were generated. The raw sequencing reads from the two platforms were combined, and reads of quality score below 30 were trimmed using CLC Genomics Workbench 7.03 (http://www.clcbio.com/products/clc-genomics-workbench). They were assembled into contigs using the default parameters.

### Structural analysis of genome

The BLASTN [51] program was used to analyze the assembled contigs with data in GenBank. Contigs from contaminating organisms were removed using a threshold e-value of 1e-10 and manual inspections of the sequence coverages. BUSCO [52] was used to search the 429 core eukaryotic orthologs within genomes of *T. gondii*, *E. tenella* and *C. cayetanensis* and assess the completeness of the genome sequencing. Simple tandem repeat and low complexity sequences in the *C. cayetanensis* genome were identified using RepeatMasker version 4.0.3 (http://repeatmasker.org/), whereas LTR-retrotransposons were identified using LTRharvest [53]. Circos [54] was used to present the alternating patterns of repeat-rich and repeat-poor sequences in long contigs.

All predicted LTR-retrotransposons were extracted and translated into amino acid sequences. HMMER (http://hmmer.janelia.org/) was used to search chromodomain (PF00385) and reverse transcriptase (PF00078) motifs in these sequences using the HMM model from Pfam [55] (http://pfam.xfam.org/). A cluster analysis of all LTR-retrotransposons was conducted based on nucleotide sequence identities. The longest LTR-retrotransposon from the biggest group was used for phylogenetic analysis. The chromovirus-type LTR sequence of *E. tenella* was randomly chosen and other LTR-retrotransposons were retrieved from NCBI GenBank. ClustalX v2 [56] was used in the preparation of a sequence alignment of LTR retrotransposons and MEGA v6 [57] was used in the construction of a neighbor-joining tree with the maximum composite likelihood mode for distance calculation and 1000 replications for bootstrapping.

Two command line software packages, tRNAscan-SE v1.3.1 [58] and ARAGORN v1.2.36 [59], were used to identify tRNA genes in the *C. cayetanensis* genome. Both of them were executed using the default settings and the general tRNA model or standard genetic codon, with the final results combined. Ribosomal RNA genes were identified using RNAmmer v1.2 [60]. Other genomic features were identified using in-house scripts.

### Gene prediction and functional annotation

Protein-encoding genes in the *C. cayetanensis* genome were predicted using a pipeline of three software packages, including AUGUSTUS v2.7 [61], SNAP [62], and GeneMark-ES [63]. AUGUSTUS and SNAP were trained with the gene-model of *E. tenella* (ToxoDB release-11.0), while GeneMark-ES is a self-training gene predictor. After examination of outcomes of gene predictions (data not shown), we kept all genes predicted by AUGUSTUS, because they fit well into the gene model. New genes predicted by both SNAP and GeneMark-ES were combined with the results of AUGUSTUS as the final protein-coding gene set of *C. cayetanensis*. SignalP v4.1 [64] and TMHMM v2.0 [65] with default settings were used to identify signal peptides and transmembrane domains within the predicted proteins, respectively. Proteins targeting the apicoplast were predicted using ApicoAP [66]. GPI anchor attachment signals were identified using the GPI-SOM webserver [67].

### Metabolism and invasion-related protein analysis

A BLASTP [51] search of the GenBank NR database and a webserver KAAS [68] were used to map the predicted proteins to specific cellular metabolic pathways. We consider the parasite to possess a certain pathway if it has the gene encoding the essential enzymes for it. The comparison of metabolism among apicomplexans was based on these analytic results and data from public databases LAMP (Library of Apicomplexan Metabolic Pathways, release-2) [12] and EuPathDB (http://eupathdb.org/eupathdb/).

Orthologs of other apicomplexans in the predicted proteome of *C. cayetanensis* were identified by using OrthoMCL [69]. Groups of paralogs within the genome were also identified by inspection of the results. The potential functions of the largest group of paralogs were identified through BLASTP analysis against the GenBank database. The identification of apical complex proteins and protein domains were conducted using the webserver Pfam [55]. Venn diagrams of protein domains shared by five apicomplexans were drawn by using the Venny tool (http://bioinfogp.cnb.csic.es/tools/venny/index.html). The phylogenetic relationship between *C. cayetanensis* and common apicomplexans was assessed by neighbor-joining analysis of an alignment of concatenated protein sequences of 100 orthologs, as described by Woo [70]. Gblocks [71] was used to remove the highly divergent regions before the construction of the phylogenetic tree.

A comparison of transporter proteins was conducted based on the Pfam search results. The database for coccidia-specific rhoptry kinases and pseudokinases HMM profiles [40], which classifies ROPKs from the genomes of *T. gondii*, *N. caninum*, *E. tenella*, and other apicomplexans into 42 distinct subfamilies, was used in the prediction and analysis of ROPKs in *C. cayetanensis* with the best hit score threshold set at 100. All putative ROPKs sequences identified in *E. tenella*, *E. falciformis*, and *C. cayetanensis* were extracted and analyzed with the neighbor-joining method described above.

## Ethics approval and consent to participate

The genome sequencing was done on a delinked residual diagnostic specimen. The work was covered by Human Subjects Protocol No. 990115 “Use of residual human specimens for the determination of frequency of genotypes or sub-types of pathogenic parasites,” which was reviewed and approved by the Institutional Review Board of the Centers for Disease Control and Prevention (CDC). No personal identifier was associated with the specimen at the time of its submission for diagnostic service at CDC.

## Availability of data and materials

The datasets supporting the conclusion of this article, including all Sequence Read Archive (SRA) data (SRX665300 and SRX681889), assembled contigs (ASM76915v1), and annotations (JROU00000000) are available in the NCBI BioProject under the accession No. PRJNA256967. The phylogenetic data supporting the conclusions of this article are available in the TreeBase (http://purl.org/phylo/treebase/phylows/study/TB2:S19120).

## Additional files

Additional file 1: Figure S1.
*De novo* assembly of *Cyclospora cayetanensis*. A total of 4,811 contigs with an overall length of 46,816,962 bp, mean length of 9,713 bp, and N50 contig length of 55,741 bp, were generated in the *de novo* assembly of sequences. (DOCX 71 kb) Additional file 2: Table S1. Summary of *Cyclospora cayetanensis* genome. (DOCX 14 kb) Additional file 3: Table S2. Assessment of the completeness of sequenced *Toxoplasma gondii*, *Eimeria tenella* and *Cyclospora cayetanensis* genomes based on core eukaryotic protein-encoding genes search using BUSCO. (DOCX 14 kb) Additional file 4: Figure S2. Predicted LTR-retrotransposons in *Cyclospora cayetanensis*. A total of 87 LTR-retrotransposons were detected in *C. cayetanensis*. The x-axis represents the length of LTR-retrotransposons, and y-axis represents the average lengths of upstream and downstream LTRs of each retrotransposon. The darkness represents the sequence similarities between the upstream and downstream LTRs of each retrotransposon. (DOCX 83 kb) Additional file 5: Figure S3. Evolutionary relationship of LTR-retrotransposons based on neighbor-joining analysis using genetic distances calculated with the maximum composite likelihood method. The LTR-retrotransposon in *Eimeria tenella* is placed within the clade formed by chromoviruses which are widely present in eukaryotic genomes. The LTR-retrotransposon of *Cyclospora cayetanensis* is clearly out of the clade. Numbers on branches are percent bootstrap values >50 from 1,000 replications. (DOCX 58 kb) Additional file 6: Table S3. Comparison of essential cellular metabolic pathways among some common apicomplexan parasites. (XLSX 70 kb) Additional file 7: Table S4. Comparison of host cell invasion-related adhesins among *Toxoplasma gondii*, *Eimeria tenella*, and *Cyclospora cayetanensis.* (DOCX 29 kb) Additional file 8: Table S5. Members of a major group of paralogs detected in the *Cyclospora cayetanensis* genome and results of BLASTP against the GenBank protein database. (XLSX 33 kb) Additional file 9: Table S6. Comparison of host cell invasion-related peptidases and proteases among *Toxoplasma gondii*, *Eimeria tenella*, and *Cyclospora cayetanensis.* (DOCX 24 kb) Additional file 10: Table S7. Comparison of host cell invasion-related protein phosphatases, kinases, and other signaling related proteins among *Toxoplasma gondii*, *Eimeria tenella*, and *Cyclospora cayetanensis.* (DOCX 25 kb)

## Abbreviations

AMA1: apical membrane antigen 1
CoA: coenzyme A
EMP1: erythrocyte membrane protein 1
GPI: glycosylphosphatidylinositol
GRA: dense granule protein
ICDH: isocitrate dehydrogenase
LA: lipoic acid
LTR: long terminal repeat
MIC: microneme protein
PEP: phosphoenolpyruvate
PLP: pyridoxal 5-phosphate
PRPP: phosphoribosyl pyrophosphate
PV: parasitophorous vacuole
RON: rhoptry neck protein
ROP: rhoptry protein
ROPK: rhoptry protein kinases
SAG: surface antigen
SRS: SAG1-related sequences
STR: short tandem repeat
TCA: tricarboxylic acid
THF: tetrahydrofolate
