# Supplementary material for: Comparative genomics reveals Cyclospora cayetanensis possesses coccidia-like metabolism and invasion components but unique surface antigens
Source: BMC Genomics. 2016 Apr 30;17:316. doi: 10.1186/s12864-016-2632-3 (PMC4851813; doi:10.1186/s12864-016-2632-3)

**Additional file 1: Figure S1**. ***De novo* assembly of *Cyclospora cayetanensis*.** A total of 4,811 contigs with an overall length of 46,816,962 bp, mean length of 9,713 bp, and N50 contig length of 55,741 bp, were generated in the *de novo* assembly of sequences.


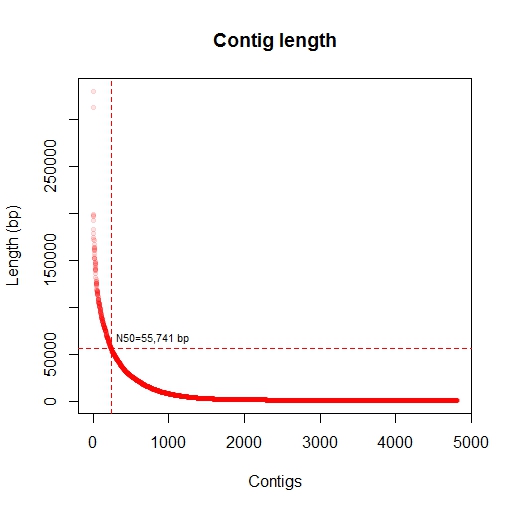

Supplement: Additional file 1: Figure S1. — De novo assembly of Cyclospora cayetanensis. A total of 4,811 contigs with an overall length of 46,816,962 bp, mean length of 9,713 bp, and N50 contig length of 55,741 bp, were generated in the de novo assembly of sequences. (DOCX 71 kb) [file 12864_2016_2632_MOESM1_ESM.docx]
