# Supplementary material for: Comparative genomics reveals Cyclospora cayetanensis possesses coccidia-like metabolism and invasion components but unique surface antigens
Source: BMC Genomics. 2016 Apr 30;17:316. doi: 10.1186/s12864-016-2632-3 (PMC4851813; doi:10.1186/s12864-016-2632-3)
Supplement: Additional file 2: Table S1. — Summary of Cyclospora cayetanensis genome. (DOCX 14 kb) [file 12864_2016_2632_MOESM2_ESM.docx]

**Additional file 2: Table S1. Summary of *Cyclospora cayetanensis* genome**

|  | Raw assembly | Draft genome* |
| --- | --- | --- |
| Total length (bp) | 46,816,962 | 44,034,550 |
| No. of contigs | 4,811 | 2,297 |
| Maximum contig length (bp) | 330,455 | 330,455 |
| Mean contig length (bp) | 9,713 | 19,170 |
| N50 (bp) | 55,741 | 61,202 |
| N90 (bp) | 5,449 | 11,522 |

*After elimination of contaminants from bacteria, Archaea, and host DNA.
