# Supplementary material for: Comparative genomics reveals Cyclospora cayetanensis possesses coccidia-like metabolism and invasion components but unique surface antigens
Source: BMC Genomics. 2016 Apr 30;17:316. doi: 10.1186/s12864-016-2632-3 (PMC4851813; doi:10.1186/s12864-016-2632-3)
Supplement: Additional file 3: Table S2. — Assessment of the completeness of sequenced Toxoplasma gondii, Eimeria tenella and Cyclospora cayetanensis genomes based on core eukaryotic protein-encoding genes search using BUSCO. (DOCX 14 kb) [file 12864_2016_2632_MOESM3_ESM.docx]

**Additional file 3: Table S2. Completeness of genomes of *Toxoplasma gondii*, *Eimeria tenella* and *Cyclospora cayetanensis* based on eukaryotic core protein-coding genes search using BUSCO software.**

| Species | Total BUSCO groups | Complete single-copy BUSCOs | Complete duplicated BUSCOs | Fragmented BUSCOs | Missing BUSCOs |
| --- | --- | --- | --- | --- | --- |
| *T. gondii* | 429 | 338 (78.8%) | 65 (15.2%) | 27 (6.3%) | 64 (14.9%) |
| *E. tenella* | 429 | 207 (48.3%) | 28 (6.5%) | 85 (19.8%) | 137 (31.9%) |
| *C. cayetanensis* | 429 | 229 (53.4%) | 34 (7.9%) | 90 (21.0%) | 110 (25.6%) |
