# Supplementary material for: Comparative genomics reveals Cyclospora cayetanensis possesses coccidia-like metabolism and invasion components but unique surface antigens
Source: BMC Genomics. 2016 Apr 30;17:316. doi: 10.1186/s12864-016-2632-3 (PMC4851813; doi:10.1186/s12864-016-2632-3)

**Additional file 4: Figure S2. Predicted LTR-retrotransposons in *Cyclospora cayetanensis*.** A total of 87 LTR-retrotransposons were detected in *C. cayetanensis*. The x-axis represents the length of LTR-retrotransposons, and y-axis represents the average lengths of upstream and downstream LTRs of each retrotransposons. The darkness represent the sequence similarities between the upstream and downstream LTRs of each retrotransposons.


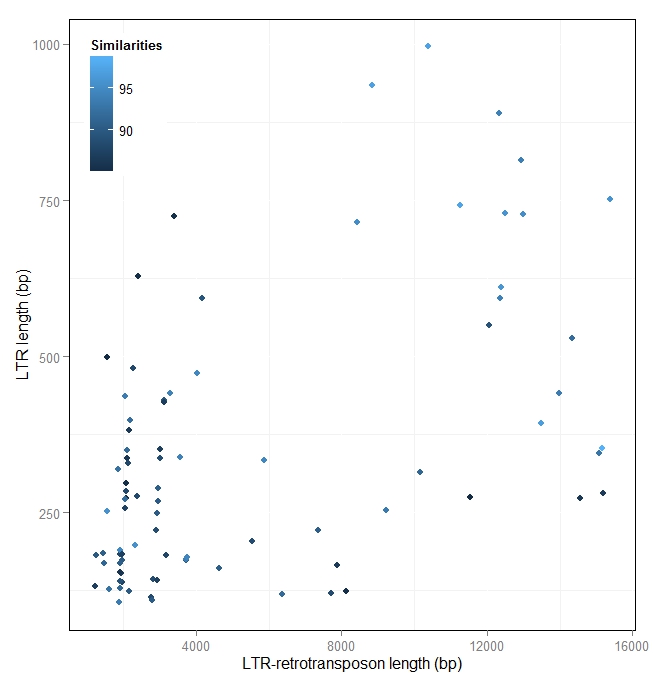

Supplement: Additional file 4: Figure S2. — Predicted LTR-retrotransposons in Cyclospora cayetanensis. A total of 87 LTR-retrotransposons were detected in C. cayetanensis. The x-axis represents the length of LTR-retrotransposons, and y-axis represents the average lengths of upstream and downstream LTRs of each retrotransposon. The darkness represents the sequence similarities between the upstream and downstream LTRs of each retrotransposon. (DOCX 83 kb) [file 12864_2016_2632_MOESM4_ESM.docx]
