# Supplementary material for: Comparative genomics reveals Cyclospora cayetanensis possesses coccidia-like metabolism and invasion components but unique surface antigens
Source: BMC Genomics. 2016 Apr 30;17:316. doi: 10.1186/s12864-016-2632-3 (PMC4851813; doi:10.1186/s12864-016-2632-3)
Supplement: Additional file 5: Figure S3. — Evolutionary relationship of LTR-retrotransposons based on neighbor-joining analysis using genetic distances calculated with the maximum composite likelihood method. The LTR-retrotransposon in Eimeria tenella is placed within the clade formed by chromoviruses which are widely present in eukaryotic genomes. The LTR-retrotransposon of Cyclospora cayetanensis is clearly out of the clade. Numbers on branches are percent bootstrap values >50 from 1,000 replications. (DOCX 58 kb) [file 12864_2016_2632_MOESM5_ESM.docx]

**Additional file 5: Figure S3.** **Evolutionary relationship of LTR-retrotransposons based on neighbor-joining analysis using genetic distances calculated with the maximum composite likelihood method.** The LTR-retrotransposon in *Eimeria tenella* is placed within the clade formed by chromoviruses which are widely present in eukaryotic genomes. The LTR-retrotransposon of *Cyclospora cayetanensis* is clearly out of the clade. Numbers on branches are percent bootstrap values >50 from 1,000 replications

MarY1 AB028236.1

Sushi-ichi AF030881.2

Pyret AB062507.1

Amn-ichi FJ158990.1

Maggy L35053.1

Pyggy AF533703.1

**Eten_Chromo_HG674968**

PT-Galadriel GQ294570.1

PT1-Reina GQ294568.1

Osvaldo AJ133521.1

Tv1 AF056940.1

Micropia-Dm11 X14037.1

SURL AJ621597.1

Mdg3 X95908.1

Skipper AF049230.1

**Ccay_LTR18**

Tor4a AY634220.1

HMS-Beagle HM214799.1

Mdg1 X59545.1

Tor2 AY634223.1

blastopia JQ666294.1

Tor1 AY634221.1

Kabuki AB032718.1

CsRn1 AY013571.1

Boudicca AY662653.1

80

100

89

55

54

76

54

50

51

56

0.2

Chromoviruses
