# Supplementary material for: Comparative genomics reveals Cyclospora cayetanensis possesses coccidia-like metabolism and invasion components but unique surface antigens
Source: BMC Genomics. 2016 Apr 30;17:316. doi: 10.1186/s12864-016-2632-3 (PMC4851813; doi:10.1186/s12864-016-2632-3)
Supplement: Additional file 7: Table S4. — Comparison of host cell invasion-related adhesins among Toxoplasma gondii, Eimeria tenella, and Cyclospora cayetanensis. (DOCX 29 kb) [file 12864_2016_2632_MOESM7_ESM.docx]

**Additional file 7: Table S4.** **Comparison of host cell invasion-related adhesins among *Toxoplasma gondii*, *Eimeria tenella*, and *Cyclospora cayetanensis****

|  |  |  |  |  |
| --- | --- | --- | --- | --- |
| **Domain family** | **Protein/gene ID** | ***T. gondii*** | ***E. tenella*** | ***C. cayetanensis*** |
| AMA1 | AMA1 | TM+2xAMA1 | SP+TM+AMA1 | 2TM+2xAMA1 |
|  | AMA2 | TM+AMA1 | SP+TM+AMA1 | SP+2TM+AMA1 |
|  | AMA3 | SP+TM+AMA1 | SP+TM+AMA1 | TM+AMA1 |
|  | AMA1-like | SP+2TM+EGF2+2xhEGF | TM+AMA1+2xhEGF | SP+2TM+AMA1+4xhEGF |
| EGF-like | MIC7 | TM+2xEGF_CA+cEGF | TM+3xEGF_CA | TM+3xEGF_CA |
|  | MIC12 | SP+20xEGF_CA+cEGF+5xEGF3 | SP+20xEGF_CA+cEGF+2xEGF3 | SP+19xEGF_CA+cEGF+4xEGF3 |
|  | MIC12-like | 5xEGF_CA+cEGF+FXa_inhibition | SP+4xEGF_CA | 4TM+Ribosomal_S9+4xEGF_CA+FXa_inhibition |
|  | MIC-25 | TM+3xEGF_CA |  |  |
|  | MIC-putative | TM+EGF |  |  |
|  | MIC-putative | 4TM+Pepti_C11+2xEGF_CA | SP+Notch+EGF_CA+cEGF | 2xEGF_CA |
| MAR_Sialic | MCP4 | SP+TM+3xMAR_Sialic |  | No domain** |
|  | MCP5 |  | SP+6xMAR_Sialic | MAR_Sialic |
|  | MIC1 | SP+2xMAR_Sialic+TgMIC1 |  |  |
|  | MIC13/MCP2 | SP+3xMAR_Sialic | SP+MAR_Sialic | SP+GPI+MAR_Sialic |
|  | MIC13-like |  | SP+2xMAR_Sialic |  |
| TSP1 & Notch | MIC2 | SP+TM+6xTSP1+VWA | SP+TM+5xTSP1+VWA | 3xTSP1+VWA |
|  | MIC14 | TM+TSP1 | SP+2TM | 3xTSP1 |
|  | MIC-putative | TSP1 | SP+TM+TSP1 | TSP1 |
|  | MIC-putative | SP+TM+4xTSP1 | SP | SP+TSP1 |
|  | MIC-putative | TM+4xNotch+14xTSP1 | SP+TM+4xNotch+15xTSP1 | Notch+5xTSP1 |
|  | MIC-putative |  |  | TM+Notch+TSP1 |
|  | MIC-putative | SP+6xNotch | 6xNotch |  |
|  | MIC-putative | TM+Notch | TM+Notch |  |
| Sushi | RON1 | SP+GPI+TM+Sushi | No domain* | SP+GPI |
|  | RON9 | TM+Sushi+Ank2+Ank5 | SP+TM+GPI+Sushi+Ank2+Ank5 | SP+TM+GPI+2xAnk2+Ank5 |
| MACPF | PLP1 | MACPF | Pkinase+MACPF | MACPF |
|  | PLP1-like | 7TM | 6TM | 6TM+MACPF |
|  | PLP1-like |  | SP+MACPF |  |
|  | PLP2 | MACPF |  |  |
| CLAG | RON2 | SP+3TM+CLAG | SP+3TM | SP+4TM |
|  | RON2L1 | SP+CLAG | TM | SP+TM+CLAG |
|  | RON2L2 | SP+4TM+CLAG | SP+3TM | 2TM |
|  | RON2L3 |  | 3TM+CLAG | No domain* |
| MAM | MAM | MAM+Cu_amine_oxid | SP+TM+MAM+2xCu_amine_oxid | TM+MAM+Cu_amine_oxid |
| SAG | SRS | SP/TM+SAG (101)*** | TA4_surface_antigen (62) | TA4_surface_antigen (5) |
| CAP | SCP family | SP+TM+CAP | SP+2TM+CAP | SP+GPI+CAP |
|  | SCP family | SP+CAP | CAP | CAP |
|  | SCP family | CAP |  |  |
| PAN/Apple | MIC4 | SP+4xPAN1+2xPAN4 | SP+6xPAN1+5xPAN4 | SP+6xPAN1+5xPAN4 |
|  | MIC17A | SP+2xPAN1+PAN4+PAN3 |  |  |
|  | MIC17B | SP+2xPAN1+PAN4+PAN3 |  |  |
|  | MIC17C | SP+TM+2xPAN1+PAN4 |  |  |
|  | MIC18 | SP+6xPAN1+4xPAN4 |  |  |
|  | MIC24 | SP+3xPAN1+4xPAN4 | SP+3xPAN4 | SP+4xPAN4 |
|  | MIC-putative | SP+3xPAN1+21xPAN4 | 2xPAN1+5xPAN4 | SP+PAN3+10xPAN4 |
|  | MIC-putative | PAN1+3xPAN4 | SP+4xPAN4 | 3xPAN4 |
|  | MIC-putative | SP+PAN1+3xPAN4 |  | SP+PAN1 |
|  | MIC-putative | SP+PAN1+14xPAN4 |  | SP+GPI+3xPAN1+2xPAN4 |
|  | MIC-putative | SP+2xPAN1+PAN3+PAN4 |  |  |
|  | MIC-putative | SP+2xPAN4 |  |  |
|  | MIC-putative |  | SP+PAN4 |  |
|  | MIC-putative |  | SP+PAN1 |  |
|  | MIC-putative |  |  | SP+3xPAN1+PAN4 |
|  | MIC-putative |  |  | SP+3xPAN4 |
| Myosin | MyoA | Myosin_head | Myosin_head | Myosin_head |
| Actin | ACT1 | Actin | Actin | Actin |
| Gliding-associated protein | GAP40 | 9TM | 10TM | 10TM |
|  | GAP45 | No domain** | No domain | No domain |
|  | GAP50 | SP+2TM | SP+TM+GPI | 2TM+GPI |

- AMA1= Apical membrane antigen 1.
- EGF= Epidermal growth factor.
- hEGF= Human growth factor-like EGF.
- EGF_CA= Calcium-binding EGF domain.
- cEGF= Complement Clr-like EGF; domains have six conserved cysteine residues disulfide-bonded to form the characteristic pattern 'ababcc‘.
- FXa_inhibition= Coagulation Factor Xa inhibitory site.
- Pepti_C11= Clostripain family; it may have lost its catalytic activity yet retained its function in protein recognition and binding.
- MAR_Sialic= Sialic-acid binding micronemal adhesive repeat .
- TgMIC1= *T. gondii* micronemal protein 1 (TgMIC1).
- TSP1= Thrombospondin, TRAP proteins of *Plasmodium*; it is involved in cell-cell interaction.
- Notch= LNR (Lin-12/Notch repeat) domain is found in three tandem copies in Notch related proteins, acting as a transmembrane receptor for intercellular signals that specify cell fates during animal development.
- VWA= Von Willebrand factor type A domain.
- Kringle= Kringles are found in blood clotting and fibrinolytic proteins. Kringle domains are believed to play a role in binding mediators (e.g., membranes, other proteins or phospholipids), and in the regulation of proteolytic activity.
- PAN= It contains a conserved core of three disulphide bridges and may mediate protein-protein or protein-carbohydrate interactions.
- Sushi= It may be one type of selectins, which are a family of cell adhesion molecules (CAMs). It is localized to the rhoptry neck in *T. gondii*, and may be essential for invasion.
- Ank= Ankyrin repeat domains, one of the most common protein-protein interaction platform in nature.
- MACPF= MAC-perforin domain, membrane attack complex (MAC) proteins of complements (C6, C7, C8α, C8β and C9) and perforins (PF). Many members of this protein family are important pore forming toxins in eukaryotes.
- CLAG= Cytoadherence-linked asexual protein is a *Plasmodium* surface protein that has been shown to be involved in the binding of *Plasmodium falciparum* infected erythrocytes to host endothelial cells, in a process called cyto-adherence.
- MAM= An extracellular domain found in many receptors and is thought to have an adhesive function.
- Cu_amine_oxid= Copper amine oxidases. MAM+Cu_amine_oxid may oxidize amine side chains of basic amino acids such as lysine in extracellular proteins. Lysyl oxidases are important in biogenesis of fibrillar extracellular matrices.
- SAG= SAG1-related surface protein, which is present on the surface of *Toxoplasma* linked with glycosylphosphatidylinositol (GPI) anchor.
- Surface_antigen= Sporozoite TA4 surface antigen, which is common in *E. tenella.*
- CAP= Cysteine-rich secretory protein family, involved in processes including the regulation of extracellular matrix and branching morphogenesis, and in cell-cell adhesion during fertilization.
- Myosin_head= Motor domain. Myosin interacts with actin.
- Actin= Actin, major components of the cytoskeleton.

*Most of the descriptions of the functional domains were based on search of the PFAM database 27.0 (March 2013) <http://pfam.xfam.org/>.

**Cells with “No domain” represent that there is a lineage specific orthologous protein in this apicomplexan but with no functional domains.

***Numbers in parentheses represent numbers of genes that contain the protein domains.
