# Supplementary material for: Comparative genomics reveals Cyclospora cayetanensis possesses coccidia-like metabolism and invasion components but unique surface antigens
Source: BMC Genomics. 2016 Apr 30;17:316. doi: 10.1186/s12864-016-2632-3 (PMC4851813; doi:10.1186/s12864-016-2632-3)
Supplement: Additional file 9: Table S6. — Comparison of host cell invasion-related peptidases and proteases among Toxoplasma gondii, Eimeria tenella, and Cyclospora cayetanensis. (DOCX 24 kb) [file 12864_2016_2632_MOESM9_ESM.docx]

**Additional file 9: Table S6.** **Comparison of host cell invasion-related peptidases and proteases among *Toxoplasma gondii*, *Eimeria tenella*, and *Cyclospora cayetanensis****

|  | |  | |  | |  |  |
| --- | --- | --- | --- | --- | --- | --- | --- |
| **Domain family** | **Protein/gene ID** | | ***T. gondii*** | | ***E. tenella*** | | ***C. cayetanensis*** |
| Serine proteases | SUB1 | | SP+GPI+Pepti_S8 | |  | |  |
|  | SUB2 | | SP+2TM+Pepti_S8 | | TM+Pepti_S8 | | 2TM+Pepti_S8 |
|  | SUB2-like | |  | |  | | 2TM+Pepti_S8 |
|  | SUB3 | | TM+Pepti_S8 | |  | |  |
|  | SUB4 | | SP+TM+Pepti_S8 | | SP+Pepti_S8 | | Pepti_S8 |
|  | SUB5 | | Pepti_S8 | | Pepti_S8 | | No domain** |
|  | SUB6 | | SP+GPI+Pepti_S8 | | Pepti_S8 | | Pepti_S8 |
|  | SUB6-like | |  | | SP+Pepti_S8 | | Pepti_S8 |
|  | SUB7 | | SP+TM+Pepti_S8 | |  | |  |
|  | SUB8 | | Peptidase_S8 | | No domain | | Pepti_S8 |
|  | SUB8-like | |  | | SP+Pepti_S8 | |  |
|  | SUB9 | | Pepti_S8 | | TM+Pepti_S8 | | SP+2xPepti_S8 |
|  | SUB10 | | TM+Pepti_S8 | |  | |  |
|  | SUB11 | | Pepti_S8+2xEGF_CA | | SP+Pepti_S8+2xEGF_CA | | SP+GPI+Pepti_S8+2xEGF_CA |
|  | SUB-putative | |  | | TM+Pepti_S8 | |  |
|  | SUB-putative | |  | | SP+Pepti_S8 | |  |
| Cysteine proteases | CPB | | SP+TM+Pepti_C1 | | SP+Pepti_C1 | | SP+Pepti_C1 |
|  | CPL | | TM+Pepti_C1+Inhibitor_I29 | | Pepti_C1 | | TM+Pepti_C1+Inhibitor_I29 |
|  | CPC1 | | SP+TM+2xPepti_C1 | | SP+2xPepti_C1 | | 2xPepti_C1 |
|  | CPC2 | | SP+Pepti_C1+CathepsinC_exc | | Pepti_C1 | | TM+Pepti_C1 |
|  | CPC3 | | SP+Pepti_C1+CathepsinC_exc | | Pepti_C1 | |  |
| Insulinases | TLN1 | | SP+TM+Pepti_M16+Pepti_M16C | | SP+Pepti_M16_C | | Pepti_M16+Pepti_M16_C |
|  | TLN1-like | | SP+Pepti_M16+2xPepti_M16C | | SP+Pepti_M16+2xPepti_M16C | | TM+Pepti_M16+2xPepti_M16_C |
|  | TLN1-like | | SP+Pepti_M16+2xPepti_M16C | | Pepti_M16+2xPepti_M16C | | Pepti_M16+2xPepti_M16_C |
|  | TLN1-like | | SP+Pepti_M16+2xPepti_M16C | |  | |  |
|  | TLN1-like | | SP+Pepti_M16+Pepti_M16C | | SP+Pepti_M16+Pepti_M16C | | Pepti_M16+Pepti_M16_C |
|  | TLN1-like | | SP+GPI+Pepti_M16+Pepti_M16C | | SP+Pepti_M16+Pepti_M16C | | Pepti_M16+Pepti_M16_C |
|  | TLN1-like | | Pepti_M16+Pepti_M16C | | SP+Pepti_M16C | | Pepti_M16+Pepti_M16_C |
|  | TLN1-like | | SP+Pepti_M16C+M16C_assoc | | SP+Pepti_M16C | | TM+Pepti_M16_C |
|  | TLN1-like | |  | | TM+Pepti_M16 | |  |
|  | TLN4 | | Pepti_M16+2xPepti_M16_C | | SP | | Pepti_M16 |
| Rhomboid | ROM1 | | 6TM+Rhomboid | | 7TM+Rhomboid | | 6TM+Rhomboid |
|  | ROM2 | | 7TM+Rhomboid | |  | | 6TM+GPI+RRM1+Rhomboid |
|  | ROM3 | | 8TM+Rhomboid | | 4TM+Rhomboid | |  |
|  | ROM4 | | SP+6TM+Rhomboid | | 6TM+Rhomboid | | 6TM+Rhomboid |
|  | ROM5 | | 6TM+Rhomboid | | 5TM+Rhomboid | | Rhomboid |
|  | ROM6 | | Rhomboid | |  | | Rhomboid |

- Pepti_S8= Subtilases, which are a family of subtilisin-like serine proteases.
- Pepti_C1= Cysteine proteases, also known as thiol proteases, are enzymes that degrade proteins. They are localized to the rhoptries and are involved in the processing of rhoptry proteins. In PV, they may be involved in degrading host proteins.
- Inhibitor_I29= Protease inhibitors, which can inhibit the function of proteases.
- CathepsinC_exc= dipeptidyl peptidase I (DPP-I) is a lysosomal exo-cysteine protease belonging to the peptidase C1 family.
- Pepti_M16= Insulinase.
- Pepti_M16_C= Peptidase M16 inactive domain. Peptidase M16 consists of two structurally like domains. One is the active peptidase, whereas the other is inactive. The two domains hold the substrate like a clamp.
- M16C_assoc= Peptidase M16C associated, which tends to be found near the C-terminus of the metalloprotease M16C.
- Rhomboid= Rhomboid proteases, a family of intramembrane proteases that exist in almost all organisms. They irreversibly cleave the transmembrane domains of other transmembrane proteins.

*Most of the descriptions of the functional domains were based on the search of the PFAM database 27.0 (March 2013) <http://pfam.xfam.org/>.

**Cells with “No domain” represent that there is a lineage specific orthologous protein in this apicomplexan but with no functional domains.
