# Supplementary material for: Comparative genomics reveals Cyclospora cayetanensis possesses coccidia-like metabolism and invasion components but unique surface antigens
Source: BMC Genomics. 2016 Apr 30;17:316. doi: 10.1186/s12864-016-2632-3 (PMC4851813; doi:10.1186/s12864-016-2632-3)
Supplement: Additional file 10: Table S7. — Comparison of host cell invasion-related protein phosphatases, kinases, and other signaling related proteins among Toxoplasma gondii, Eimeria tenella, and Cyclospora cayetanensis. (DOCX 25 kb) [file 12864_2016_2632_MOESM10_ESM.docx]

**Additional file 10: Table S7.** **Comparison of host cell invasion-related protein phosphatases, kinases, and other signaling related proteins among *Toxoplasma gondii*, *Eimeria tenella*, and *Cyclospora cayetanensis****

|  | | |  |  |
| --- | --- | --- | --- | --- |
| **Domain family** | **Protein/gene ID** | ***T. gondii*** | ***E. tenella*** | ***C. cayetanensis*** |
| PP2C | PP2C-hn | PP2C-hn |  |  |
|  | PP2C-hn-like | SP+PP2C (10)** | SP+PP2C (4) | SP+PP2C (1) |
| Rhoptry protein kinases | ROPK | SP/TM+Pkinase (14) | SP/TM+Pkinase (18) | SP/TM+Pkinase (7) |
|  | ROP2-like | SP/TM+Kinase_like (27) | SP/TM+Kinase_like (6) | SP/TM+Kinase_like (1) |
| LMF1 | ROP14 | 11TM+LMF1 | SP+5TM+LMF1 | 8TM+LMF1 |
|  | ROP14B | 7TM+LMF1 |  | No domain*** |
|  | ROP48 | 9TM+LMF1 |  |  |
|  | ROP50 | 7TM+LMF1 | LMF1 | 3TM+2xLMF1 |
| Ion channel | Ion-channel | 4xKelch1+Kelch4+BTB2 | SP+2xKelch1+Kelch4 | 5xKelch1+Kelch4+BTB2 |
|  | Ion-channel | zf-CCCH+BTB2 | BTB2 |  |
|  | Ion-channel | SP+BTB2 |  |  |
|  | Ion-channel | 6TM+BK_channel_a+Ion_trans_2 | SP+2TM+Ion_trans_2 | 9TM+BK_channel_a+Ion_trans_2 |
|  | Ion-channel | SP+8TM+BK_channel_a+Ion_trans_2 |  | 2TM+BK_channel_a |
|  | Ion-channel | 6TM+Ion_trans | 4TM | 6TM+Ion_trans |
|  | Ion-channel | 3TM+Ion_trans+Guanylate_cyc | Guanylate_cyc | 3TM+Ion_trans+Guanylate_cyc |
|  | Ion-channel | SP+10TM+Ion_trans | 9TM+Ion_trans | 9TM+Ion_trans |
|  | Ion-channel | 20TM+4xIon_trans | 19TM+3xIon_trans | 21TM+4xIon_trans |
|  | Ion-channel | 3TM+Ion_trans | 3TM | 3TM |
|  | Ion-channel | TM+Ion_trans+PDEase_I |  |  |
| PPX1-like phosphatases | NTPase I | SP+GDA1_CD39 |  |  |
|  | NTPase II | SP+GDA1_CD39 |  |  |
|  | PPX1-like | SP+GDA1_CD39 | SP+TM+GDA1_CD39 | SP+TM+GDA1_CD39 |
|  | PPX1-like | SP+2xGDA1_CD39 | SP+GDA1_CD39+Collagen | GDA1_CD39 |
|  | PPX1-like | 2TM+2xGDA1_CD39 | SP+TM | TM+2xGDA1_CD39 |
|  | PPX1-like | SP+GDA1_CD39 |  |  |
|  | PPX1-like | SP+TM+GDA1_CD39 |  |  |
|  | PPX1-like | SP+GDA1_CD39 |  |  |
| Serine protease inhibitor | TgPI-1 | SP+4xKazal_1 |  |  |
|  | TgPI-2 | SP+3xKazal_1 |  |  |
|  | TgPI-putative | SP+7xKazal_1+6xKazal_2 |  |  |
|  | TgPI-putative | SP+2xKazal_1+Kazal_2 |  |  |
|  | TgPI-putative | SP+Kazal_1+Kazal_2 |  |  |
|  | TgPI-putative |  | TM+Kazal_1+Kazal_2 |  |

- PP2C= Protein phosphatase 2C is a Mn++ or Mg++ dependent protein serine/threonine phosphatase. PP2C-hn, rhoptry protein that is secreted into host nucleus and may target phosphorylated host nuclear proteins. Other secreted PP2C proteins may similarly target host proteins. Only secretory PP2C-hn-like proteins with signal peptides are included in the table.
- Pkinase= Protein kinase domain, a structurally conserved protein domain containing the catalytic function of protein kinases.
- Kinase-like= Kinase-like proteins. This protein family includes the pseudokinases ROP2 and ROP8 from *T. gondii*. These proteins have a typical bilobe protein kinase fold, but lack catalytic activity.
- PH= Pleckstrin homology domain, involved in intracellular signaling or as constituents of the cytoskeleton.
- EDR1= Ethylene-responsive protein kinase Le-CTR1.
- LRR_6= Leucine-rich repeat.
- LMF1= Lipase maturation factor. Lipoprotein lipase and hepatic lipase require LMF1 to fold into their active states.
- Kelch= Kelch motif.
- BTB2= Potassium channel tetramerization domain.
- Zf-CCCH= Zinc finger of the C-x8-C-x5-C-x3-H type (and similar sequences).
- BK_channel_a= BK channels (Big Potassium), also called Maxi-K or slo1, are potassium channels characterized by their large conductance of potassium ions (K+) through cell membranes.
- Ion_trans= Transmembrane ion channel family is defined in InterPro and Pfam as the family of tetrameric sodium, potassium, and calcium ion channels.
- Guanylate_cyc= Adenylate and Guanylate cyclase catalytic domain. K-channel+Cyclase+TPR, ancient alveolate specific protein. May regulate the “extrusosome” using ion flux and cyclic nucleotides.
- PDEase_I= 3'5'-cyclic nucleotide phosphodiesterases (EC 3.1.4.17), cyclic 3',5'-mononucleotide phosphodiesterase, PDE.
- GDA1_CD39= A number of nucleoside diphosphate and triphosphate hydrolases. Might catalyze the hydrolysis of extracellular nucleotides or phosphorylated small molecules and may affect host signaling.
- Collagen= Collagen helix.
- Kazal= Kazal-type serine protease inhibitor domain, usually indicative of serine protease inhibitors. However, kazal-like domains are also seen in the extracellular part of agrins, which are not known as protease inhibitors.

*Most of the descriptions of the functional domains were based on search of the PFAM database 27.0 (March 2013) <http://pfam.xfam.org/>

**Numbers in parentheses represent numbers of genes that contain the protein domains.

***Cells with “No domain” represent that there is a lineage specific orthologous protein in this apicomplexan but with no functional domains.
